# Vestigial-like 1 is a shared targetable cancer-placenta antigen expressed by pancreatic and basal-like breast cancers

**DOI:** 10.1038/s41467-020-19141-w

**Published:** 2020-10-21

**Authors:** Sherille D. Bradley, Amjad H. Talukder, Ivy Lai, Rebecca Davis, Hector Alvarez, Herve Tiriac, Minying Zhang, Yulun Chiu, Brenda Melendez, Kyle R. Jackson, Arjun Katailiha, Heather M. Sonnemann, Fenge Li, Yaan Kang, Na Qiao, Bih-Fang Pan, Philip L. Lorenzi, Mark Hurd, Elizabeth A. Mittendorf, Christine B. Peterson, Milind Javle, Christopher Bristow, Michael Kim, David A. Tuveson, David Hawke, Scott Kopetz, Robert A. Wolff, Patrick Hwu, Anirban Maitra, Jason Roszik, Cassian Yee, Gregory Lizée

**Affiliations:** 1 Department of Melanoma Medical Oncology, UT MD Anderson Cancer Center, Houston, TX USA; 2 Department of Hematopathology, UT MD Anderson Cancer Center, Houston, TX USA; 3 Cold Spring Harbor Laboratory Cancer Center, Cold Spring Harbor, NY USA; 4 Department of Surgical Oncology, UT MD Anderson Cancer Center, Houston, TX USA; 5 Department of Breast Surgery Research, UT MD Anderson Cancer Center, Houston, TX USA; 6 Department of Systems Biology, UT MD Anderson Cancer Center, Houston, TX USA; 7 Department of Bioinformatics and Computational Biology, UT MD Anderson Cancer Center, Houston, TX USA; 8 Ahmed Center for Pancreatic Cancer Research, UT MD Anderson Cancer Center, Houston, TX USA; 9 Department of Biostatistics, UT MD Anderson Cancer Center, Houston, TX USA; 10 Department of Gastrointestinal Medical Oncology, UT MD Anderson Cancer Center, Houston, TX USA; 11 Center for Co-clinical Trials, UT MD Anderson Cancer Center, Houston, TX USA; 12 Department of Pathology, UT MD Anderson Cancer Center, Houston, TX USA; 13 Department of Immunology, UT MD Anderson Cancer Center, Houston, TX USA

**Keywords:** Biotechnology, Cancer, Cancer immunotherapy, Targeted therapies, Immunology

## Abstract

Cytotoxic T lymphocyte (CTL)-based cancer immunotherapies have shown great promise for inducing clinical regressions by targeting tumor-associated antigens (TAA). To expand the TAA landscape of pancreatic ductal adenocarcinoma (PDAC), we performed tandem mass spectrometry analysis of HLA class I-bound peptides from 35 PDAC patient tumors. This identified a shared HLA-A*0101 restricted peptide derived from co-transcriptional activator Vestigial-like 1 (VGLL1) as a putative TAA demonstrating overexpression in multiple tumor types and low or absent expression in essential normal tissues. Here we show that VGLL1-specific CTLs expanded from the blood of a PDAC patient could recognize and kill in an antigen-specific manner a majority of HLA-A*0101 allogeneic tumor cell lines derived not only from PDAC, but also bladder, ovarian, gastric, lung, and basal-like breast cancers. Gene expression profiling reveals VGLL1 as a member of a unique group of cancer-placenta antigens (CPA) that may constitute immunotherapeutic targets for patients with multiple cancer types.

## Abstract (web summary)

Cytotoxic T lymphocyte (CTL)-based immunotherapies can induce tumor regressions by targeting HLA class I-bound tumor-associated peptides. Here, the authors identified a peptide derived from Vestigial-like 1 (VGLL1) as a shared, potentially therapeutic CTL target expressed by multiple cancer types.

## Introduction

Pancreatic ductal adenocarcinoma (PDAC), the most aggressive form of pancreatic cancer, remains notorious for its poor prognosis and high mortality rate, with its overall 5-year survival rate of 8% being amongst the lowest of all cancer types^1,2^. Early detection is unusual, with 85% of patients presenting with locally advanced or metastatic disease^3^. Progress toward effective treatment has been slow and the incidence of PDAC-related deaths has continued to rise^4,5^. Despite some encouraging recent improvements in survival achieved through optimizing the sequencing of surgery and chemotherapy treatment regimens, developing new and effective therapeutic options remains a dire need for advanced-stage PDAC patients^6^.

Cytotoxic T lymphocyte (CTL)-based immunotherapies have been successful at inducing objective clinical responses in a variety of cancer types^7^. Checkpoint inhibitor (CPI) therapies that act through non-specific activation of T lymphocytes have made a significant positive impact on long-term patient survival^8^. However, the benefits of CPI have mainly been limited to highly mutated tumor types like melanoma and lung adenocarcinoma that can express a large array of potential neo-antigen peptides in the context of surface HLA molecules^5,9^. Tumor-infiltrating lymphocyte (TIL) therapy, in which individual cancer patients are re-infused with T cells expanded from their own tumors, has also shown great promise for inducing the regression of bulky tumors.^10,11^ TILs are polyclonal and can recognize both patient-specific neo-antigens as well as shared tumor-associated antigens (TAA) such as melanocyte differentiation antigens (MDA) or cancer-testis antigens (CTA)^12–14^. Targeting of individual validated HLA class I-restricted TAAs through infusion of antigen-specific endogenous T cells (ETC therapy) or genetically engineered TCR-T cells has also proven successful at inducing clinical responses in patients with melanoma and other solid cancers^15–19^.

CPI- and CTL-based immunotherapies have unfortunately not shown the same beneficial impact in treating PDAC patients^20,21^. This lack of success has been attributed to the highly immune-suppressive tumor microenvironment (TME) of PDAC, in addition to the relatively low mutational burden that contributes to a dearth of neo-antigen targets^22–25^. A number of potentially targetable HLA class I-restricted peptide antigens have been identified in PDAC, most notably those derived from carcinoembryonic antigen-related cell adhesion molecule (CEACAM), mucin 16 (MUC16), mesothelin (MSLN), and mutated *KRAS*, among others^26–30^. Although promising, therapies targeting non-mutated TAAs have faced inherent limitations, including the induction of toxicities in non-tumor tissues, low prevalence of target antigen expression, or inability to break self-tolerance mechanisms that often hinders the generation of high-affinity CTLs^20,31,32^. With limited exceptions, clinical trials targeting these antigens have yielded disappointing results, underscoring the need to identify safe, immunogenic targets that demonstrate higher prevalence in PDAC patients.

In order to expand the targetable TAA landscape of PDAC, we performed tandem mass spectrometry (MS) analysis on HLA class I-bound peptide antigens isolated from tumor specimens derived from 35 PDAC patients. We report here the discovery of a shared, HLA-A*0101-restricted peptide derived from the co-transcriptional activator Vestigial-like 1 (VGLL1) that was expressed and presented in multiple patients. VGLL1 peptide-specific CTLs isolated and expanded from PBMCs (peripheral blood mononuclear cells) of a PDAC patient robustly killed autologous tumor cells, in addition to recognizing a panel of allogeneic HLA-A*0101-positive PDAC, ovarian, gastric, bladder, lung, and basal-like breast cancer cell lines. With the exception of a mammary cell line, VGLL1 CTLs did not recognize most HLA-A*0101-expressing matched normal primary cells. Patient infusion of autologous VGLL1 CTLs resulted in no autoimmune toxicities but also failed to induce a clinical response, likely due to VGLL1 antigen loss by the metastatic tumor^33^. Comparative gene expression profiling revealed that VGLL1 is a member of a unique group of cancer-placenta antigens (CPA) that demonstrate high expression in placenta, low to absent expression in essential normal tissues, and overexpression in several different cancers. Collectively, our results show that VGLL1 is an immunogenic TAA target with strong potential for enabling CTL-mediated therapies for PDAC and multiple other cancer types.

## Results

### Immunopeptidome analysis of PDAC identifies tumor-associated peptides

**Fig. 1:**
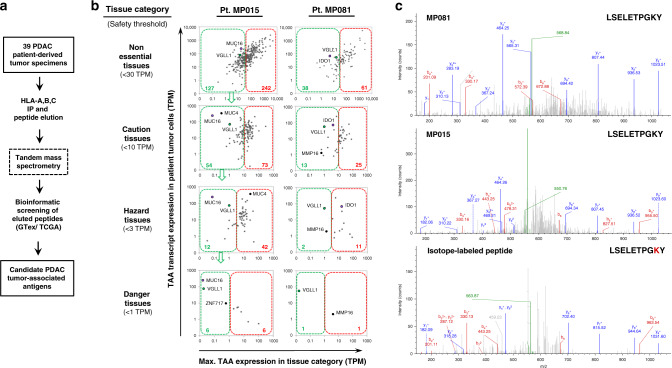
Immunopeptidome analysis reveals a VGLL1-derived peptide expressed by two PDAC patient-derived organoid lines. **a** Experimental strategy to identify PDAC tumor-specific, HLA class I-bound peptides from 39 tumor specimens derived from 35 M.D. Anderson PDAC patients. **b** Bioinformatics screening strategy to identify potentially targetable TAAs from amongst the eluted PDAC-associated peptides. Peptide-encoding genes were assessed for PDAC tumor RNAseq expression compared with transcript expression in 53 GTex Portal normal tissues. Excluding testis, normal tissues were separated into four categories (non-essential, caution, hazard, and danger tissues) that reflected the potential toxicities expected from off-tumor killing activity against different tissues (listed in Table S2). All peptide-encoding genes were filtered successively using four corresponding expression thresholds of increasing stringency (30, 10, 3, and 1 TPM, indicated by green dotted lines) to eliminate candidate TAAs most likely to elicit autoimmune toxicity in the context of CTL therapy (red dotted lines). Screening of high-confidence peptides isolated from tumor organoid cell lines of PDAC patients MP015 and MP081 is depicted, showing that few eluted peptides met these stringent criteria. **c** Mass spectra of an HLA-A*0101-restricted VGLL1-derived peptide isolated from two different PDAC organoid cell lines, MP015 and MP081 (top 2 panels). The patient-derived peptides co-eluted with and matched the MS fragmentation spectra of the synthetic isotope-labeled VGLL1 peptide LSELETPGKY (containing a ^13^C/^15^N-labeled lysine residue), with the labeled *y*^*+*^ fragment ion series (shown in blue) demonstrating an expected shift of 8 atomic mass units, unlike the *b*^+^ fragment ions (bottom panel). IP immunoprecipitation, TPM transcripts per million, TAA tumor-associated antigen.

To identify peptide targets for CTL-based immunotherapy of PDAC, we analyzed 39 tumor specimens derived from 35 PDAC patients treated at M.D. Anderson Cancer Center. This included 34 freshly-excised surgical specimens (20 metastatic and 14 primary tumors), in addition to 3 patient-derived xenografts (PDX) and 2 organoid cell lines derived from metastases. Tumor cells were lysed and subjected to total HLA class I immunoprecipitation and acid elution, followed by tandem mass spectrometry (MS) to analyze the HLA-bound peptides. Eluted peptide fragmentation spectra were searched against the SwissProt database (updated 9/2018) to identify matches encoded within the human proteome. Individual peptide matches were assessed using several orthogonal parameters, including Mascot Ion score, MS1 mass differential (delta mass), and predicted binding to the patient’s HLA allotypes as determined by high-resolution genetic sequencing^34^^,^^35^. Further validation and potential suitability as therapeutic TAA targets were determined by evaluating all peptide-encoding genes for (1) patient tumor tissue transcript expression as determined by RNAseq, (2) normal tissue transcript expression (GTex Portal database), and (3) overall expression in tumor tissues (TCGA database) (Fig. 1a) (http://www.gtexportal.org/home/, http://cancergenome.nih.gov/).

The amount of immunoprecipitated HLA class I correlated with the size of the fresh tumor specimens analyzed (*R*^2^ = 0.79), with the exception of 8 tumors (21.6%) that showed relatively low HLA class I expression as assessed by western blot analysis. Overall, the 39 tumor specimens analyzed yielded a total of 23,245 unique, high-confidence peptide identities, of which 7966 peptides (34.3%) were 8- to 13-mer peptides predicted to bind to one or more patient HLA class I allotypes. Fresh tumor specimens yielded a highly variable number of peptides, ranging from 238 to 1657 (mean = 542). For three patients, PDX derivation resulted in larger tumor specimens, yielding an increased number of eluted peptides. One of the two patient-derived organoid cell lines (MP015) yielded the highest number of eluted peptides overall (*n* = 1903), underscoring the quantitative advantage provided by expanding tumor specimens in vitro prior to MS analysis (Supplementary Table 1).

### Expression profiling identified VGLL1 as a putative pancreatic cancer TAA

To evaluate if any of the eluted peptides constituted potentially therapeutic CTL targets, peptide-encoding genes were individually assessed for normal tissue transcript expression with reference to the GTex Portal database containing RNAseq data derived from 53 different human tissues. Normal tissues (excluding testis) were categorized into four groups that reflected the potential toxicities expected from off-target killing activity by antigen-specific CTLs (Supplementary Table 2). Peptide-encoding genes were then screened using four corresponding expression filters of increasing stringency in order to eliminate candidate TAAs most likely to elicit autoimmune toxicity in the context of CTL therapy (Fig. 1b). Thus, while TAA transcript expression up to 30 TPM maximum was allowed in non-essential tissues (such as prostate, breast, and adipose tissues), a maximum expression threshold of 1 TPM was imposed for highly essential tissues such as heart and brain, for which CTL recognition can be lethal.^36,37^. Using these stringent criteria, 12 TAA peptides were deemed safest to target, the genes encoding these peptides being *MUC16* (encoding 5 unique peptides), *MUC19, ZNF717, EIF5AL1, RGPD1, SLC30A8, MIA2*, and *VGLL1* (each encoding 1 unique peptide). Peptides encoded by TAAs MSLN and IDO1 were also detected, but were excluded in the screening due to elevated RNA transcript expression in normal lung tissue (88 TPM and 16 TPM, respectively, Fig. 1b). Amongst the TAAs deemed safest to target, only 2 peptides (derived from *MIA2* and *VGLL1*) were found to be presented by tumors of more than one PDAC patient (Supplementary Table 1). A number of these same peptides, including those derived from MUC16 and IDO1, have also been reported to be expressed in the immunopeptidome of ovarian cancer specimens^38^.

The 10-mer peptide LSELETPGKY, uniquely encoded by *VGLL1*, was eluted from both PDAC patient-derived organoid cell lines MP015-Org and MP081-Org (Supplementary Data 1 and 2). This peptide was predicted to bind with high affinity to HLA-A*0101 (51 nM), and RNAseq analysis confirmed high *VGLL1* transcript expression in both organoid lines (Table 1). Peptide identity was confirmed by targeted LC-MS, in which a synthetic peptide was analyzed as part of a mixture with organoid tumor-associated peptides. As shown in Fig. 1c, the synthetic isotope-labeled peptide LSELETPGKY generated highly similar fragmentation spectra to the native VGLL1 peptide detected from PDAC organoid lines MP015-Org and MP081-Org, and was co-eluted at identical LC-MS retention times. Targeted-MS analysis on two additional HLA-A*0101-expressing cell lines (PANC10.05 and BXPC3) demonstrated that the same peptide could also be detected on PANC10.05, suggesting that LSELETPGKY might constitute a widely shared TAA (Supplementary Fig. 1).

**Table 1:** VGLL1-derived peptide eluted from two HLA-A*0101^+^ PDAC patient tumor organoids.

Patient identifier	Eluted peptides	Source gene	Match rank	Tumor RNA expression (RNAseq, TPM)	Predicted HLA-A*0101 binding affinity (nM)
MP015	LSELETPGKY	VGLL1	1	77.53	51
MP081	LSELETPGKY	VGLL1	1	56.39	51

### VGLL1 is expressed by multiple cancers and is associated with poor survival

**Fig. 2:**
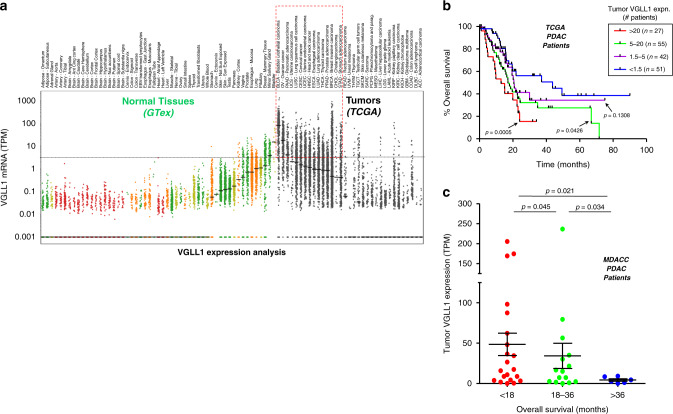
VGLL1 is overexpressed in multiple tumor types and is associated with poor pancreatic patient survival. **a** VGLL1 transcript expression in normal tissues (colored dots, GTex Portal database) and human cancers (black dots, TCGA database), as determined by RNAseq analyses. Each dot represents one normal donor or patient tumor sample. Colors correspond to the four normal tissue categories defined in Fig. 1: Green, non-essential tissues; yellow, caution tissues; orange, hazard tissues; red, danger tissues. While >95% of normal GTex caution, hazard, and danger tissue samples fell below 3 transcripts per million (TPM, dotted line), significant numbers of TCGA cancer patients demonstrated VGLL1 transcript expression above this threshold (red box). **b** Kaplan–Meier curves showing TCGA PDAC patient overall survival (OS) stratified by tumor VGLL1 transcript expression (*n* = 178). *P*-values indicate log-rank significance test results comparing the OS of three groups of VGLL1-expressing patients to those patients with low or absent VGLL1 expression using Gehan–Breslow–Wilcoxon test. **c** Patient-derived xenografts (PDX) from an independent cohort of MD Anderson metastatic PDAC patient tumors (*n* = 42) underwent RNAseq analysis after being grown in immunodeficient mice. Graph shows PDX specimens stratified into three groups corresponding to OS time and corresponding VGLL1 transcript expression. Data are presented as mean values +/− standard deviation. *P*-values indicate log-rank significance test results comparing each group using an ANOVA test. Adjustments were made for multiple comparisons.

VGLL1, previously known as TONDU, was first identified as the human homolog of the *Drosophila* Vestigial (Vg) protein, a key regulator of wing development^39,40^. Since VGLL1 is a transcriptional co-activator that binds to the TEA domain (TEAD) family of transcription factors implicated in cancer development, we examined *VGLL1* transcript expression in the 33 cancer types listed in TCGA. As shown in Fig. 2a, in comparison to most normal tissues *VGLL1* is overexpressed in several different cancers, including PDAC, bladder, ovarian, breast, lung, and stomach cancer. Interestingly, *VGLL1* appears to be preferentially expressed in basal-like breast cancers while demonstrating a relatively low prevalence in other breast cancer subtypes (Supplementary Fig. 2). A similar profile was confirmed by gene expression analysis of tumor cell lines listed in the Cancer Cell Line Encyclopedia (CCLE, Supplementary Fig. 3). According to the GTex RNAseq database, the highest median *VGLL1* transcript expression was found in four non-essential tissues: bladder (15.3 TPM), salivary gland (3.9 TPM), breast (1.3 TPM), and pituitary gland (1.0 TPM). The highest level of *VGLL1* transcript expression in essential tissues was in normal lung (1.0 TPM), esophagus (0.73 TPM), and kidney (0.34 TPM), while *VGLL1* expression in heart and brain tissues was virtually undetectable (Fig. 2a). Collectively, this data suggested that VGLL1 may constitute a safe, targetable TAA for multiple cancer types.

We next assessed if tumor *VGLL1* transcript expression was associated with cancer patient survival. As shown in Fig. 2b, TCGA PDAC patient survival (*n* = 179) was found to be inversely correlated with *VGLL1* expression: patients with high expression had a significantly shorter overall median survival compared to patients with low or absent expression (16 months vs. 37 months, *p* = 0.001). This was confirmed in an independent cohort of 42 M.D. Anderson PDAC patients for whom PDX tissues could be derived: patients showing an overall survival of less 18 months demonstrated a significantly higher mean PDX *VGLL1* expression compared to patients that survived longer than 36 months (48.5 TPM vs. 4.4 TPM, *p* = 0.003, Fig. 2c). It is worth noting that *VGLL1* transcript expression was found to be considerably higher in PDAC tumor cell lines and PDX tissues compared with surgically resected PDAC tumors, likely due to the high stromal content of many PDAC tumors in situ (Fig. 2a, c, Table 1). Highly elevated *VGLL1* expression was also associated with shorter overall survival time in breast cancer (*p* = 0.037) and stomach cancer (*p* = 0.047), but showed no association with survival in ovarian cancer (Supplementary Fig. 4)^41^. Interestingly, low or absent *VGLL1* expression was associated with shorter survival time in bladder cancer (*p* = 0.036). One potential explanation is that loss of a normal bladder tissue antigen like VGLL1 may indicate tumor dedifferentiation, which has been associated with poorer prognosis in bladder cancer and many other tumor types^42^.

### VGLL1 is a cancer-placenta antigen (CPA) with therapeutic potential

**Fig. 3:**
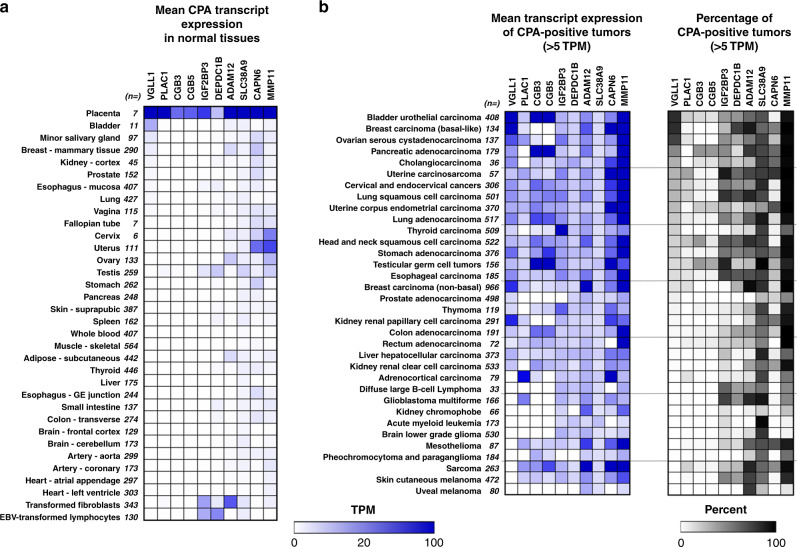
VGLL1 is a cancer-placenta antigen (CPA) demonstrating high expression in normal placenta and tumors. Gene expression profiling uncovered nine additional putative CPAs with similar expression profiles to VGLL1. **a** Heat map depicting the mean transcript expression of different CPAs in normal placenta (top), GTex normal tissues, and transformed lymphocytes and fibroblasts (bottom). Tissues are listed in order of highest to lowest VGLL1 expression, as determined by RNAseq analysis. **b** Heat maps displaying the mean CPA transcript expression (left) and frequency (right) of CPA-positive tumor specimens in 34 different TCGA cancer types as determined by RNAseq. CPA-positive specimens were defined as having tumor CPA transcript expression >5 TPM. Tumor tissues are listed in order of highest to lowest VGLL1 prevalence.

VGLL1 had been previously identified as having a regulatory role during early events in human placental development, and is a specific marker of proliferative and invasive cytotrophoblast cells^43^. In accordance with this, RNAseq gene expression data from seven human placenta samples showed that *VGLL1* demonstrated the highest expression in this tissue by a large margin (mean = 302.7 TPM), nearly 20-fold higher than its expression in normal bladder (Fig. 3a). This led us to explore the notion that cancer-placenta antigens (CPA) may constitute a distinct category of targetable TAAs analogous to cancer-testis antigens (CTAs), which have been successfully targeted with CTL-based therapies. To identify other CPAs with similar expression profiles to VGLL1, we searched the GTex, TCGA, and other RNAseq databases for genes that demonstrated the following attributes: (1) highest normal tissue expression in placenta; (2) low to absent expression in other normal tissues; and (3) elevated expression in pancreatic, breast, bladder, and/or ovarian cancer. This search yielded nine additional genes, including placenta-specific 1 (*PLAC1*), previously identified as a target of humoral antitumor immunity in cancer patients^44^. Interestingly, Chorionic Gonadotropin (CG) Beta subunits 3 and 5 (*CGB3/CGB5*), components of the CG hormone complex produced by placental trophoblasts during pregnancy, were also identified as potential CPAs due to their overexpression in a subset of pancreatic, testicular, uterine, and bladder cancers (Fig. 3b). The other 6 putative CPAs demonstrated diverse expression profiles, ranging from those found only in a restricted set of cancer types (*IGF2BP3, ADAM12*), to those overexpressed in most cancer types but also demonstrating elevated expression in normal female reproductive tissues (*CAPN6, MMP11*) (Fig. 3, Supplementary Figs. 5–15, Supplementary Tables 3–6). Although we did not detect peptides derived from these genes in this set of PDAC specimens, epitopes from several of these putative CPAs have been identified in multiple tumor types and are listed in the Immune Epitope Database (IEDB)^44^.

### VGLL1-specific CTLs were expanded from PDAC patient MP015

Patient MP015 was first diagnosed with primary PDAC in December 2011. As previously reported, two years following surgical removal of the primary pancreatic tumor, a thorascopic wedge resection of a left lung lesion was performed in November 2013 and used to derive organoid cell line MP015-Org^33^. The patient’s disease was kept in check for nearly 2 more years through a series of chemotherapeutic regimens, but following progression, he was enrolled in a cell therapy protocol approved by the M.D. Anderson Cancer Center Clinical Trials Institutional Review Board to receive autologous, expanded tumor-antigen-specific CTLs. Immunopeptidome analysis performed on the expanded organoid cell line MP015-Org in May 2015 led to the identification of 6 HLA class I-bound peptides (4 derived from MUC16 and 1 each from ZNF717 and VGLL1) that met our criteria as safe, targetable TAAs (Fig. 1b and Supplementary Table 1). Custom clinical-grade tetramers were available for 3 of the 6 potential targets: two HLA-B*3502-restricted MUC16 peptides and the single HLA-A*0101-restricted VGLL1 peptide.

**Fig. 4:**
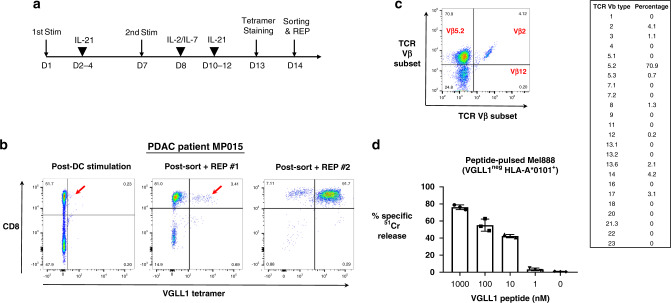
Generation of VGLL1 antigen-specific CTLs from peripheral blood of patient MP015. **a** Schematic outlining the experimental procedure for generating VGLL1-specific CD8^+^ T cells from human donor PBMCs. **b** PBMCs isolated from PDAC patient MP015 by leukapheresis were stimulated with autologous LSELETPGKY peptide-pulsed dendritic cells (DCs). After two stimulations, CD8+ and VGLL1 tetramer-positive cells were sorted and expanded using a standard rapid expansion protocol (REP). VGLL1-specific T cells were re-sorted and expanded a second time due to low numbers of antigen-specific cells following the first REP. The second REP yielded 19.6 × 10^9^ VGLL1-specific CTLs, which patient MP015 safely received as an infusion under a personalized ETC therapy Compassionate IND protocol. **c** T-cell receptor (TCR) repertoire analysis of expanded VGLL1-specific CTLs was performed using Vβ antibodies corresponding to 24 different specificities. **d** VGLL1-specific T cells expanded from patient MP015 were tested for functionality in a standard ^51^Cr release assay to assess specific lysis of Mel888 melanoma cells (VGLL1-negative HLA-A*0101-positive) pulsed with titrated amounts of LSELETPGKY peptide at a 5:1 effector-to-target (E:T) ratio. Data are presented as mean values +/− SD over three independent experiments.

Following leukapheresis, patient MP015 PBMCs were stimulated twice with individual peptide-pulsed DCs in the presence of IL-21, followed by tetramer-based sorting of antigen-specific CD8+ T cells (Fig. 4a). Although MUC16-specific CTLs failed to expand from patient PBMCs, VGLL1 CTLs expanded successfully, with VGLL1 tetramer-positive T cells comprising 3.4% of CD8+ cells after 2 weeks of DC-peptide stimulation (Fig. 4b). Cell sorting followed by employment of the rapid expansion protocol (REP) was repeated twice, resulting in nearly 20 billion expanded CTLs, of which >90% were VGLL1 tetramer-positive and demonstrated restricted Vβ usage (Fig. 4b and c). Functional VGLL1-specific CTLs were also successfully expanded from 2 of 2 healthy HLA-A*0101-positive blood donors, demonstrating the general immunogenicity of the LSELETPGKY peptide (Supplementary Fig. 16).

**Fig. 5:**
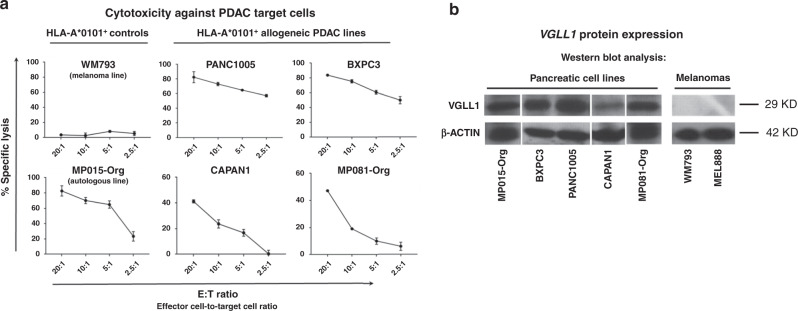
VGLL1-specific CTLs recognize and kill multiple allogeneic pancreatic cancer cell lines. **a** Expanded VGLL1-specific CD8^+^ T cells from patient MP015 were co-cultured with a panel of HLA-A*0101-positive PDAC tumor cell lines in a standard ^51^Cr release assay to measure cytotoxic activity at different effector-to-target (E:T) cell ratios. WM793 melanoma cells (VGLL1-negative, HLA-A*0101-positive) were used as a negative control line. VGLL1 CTLs robustly killed the autologous organoid cell line MP015 from which the VGLL1 peptide was originally isolated, and also demonstrated cytotoxic activity against four allogeneic, HLA-A*0101-expressing PDAC cell lines. Results show the means and standard deviations of six replicate samples, and data is representative of 4 independent experiments. **b** Western blot analysis showing expression of VGLL1 protein in all five PDAC cell lines tested was repeated three times with similar results.

Expanded CTLs from patient MP015 and Healthy Donor #1 were tested functionally using standard ^51^Cr release assays. Mel888 melanoma cells (VGLL1-negative, HLA-A*0101-positive) pulsed with titrated amounts of VGLL1 peptide elicited recognition and killing at peptide concentrations as low as 10 nM, indicating relatively high affinity for cognate peptide (Fig. 4d and Supplementary Fig. 16). Importantly, expanded patient-derived CTLs also showed robust recognition of the autologous organoid cell line MP015-Org from which the VGLL1 peptide was originally detected by MS (Fig. 5a). In October 2015 following a pre-treatment regimen of Cytoxan, patient MP015 was infused with 19.6 billion autologous, expanded VGLL1-specific CTLs, subsequently receiving interleukin-2 and pembrolizumab. Although the patient experienced a transient fever (a frequent side effect of T-cell-infusion-induced cytokine release), they experienced no adverse events indicating potential CTL-mediated toxicities. Unfortunately, scans in late November 2015 showed rapid disease progression manifested as an interval increase in lung lesions and pleural-based metastatic disease^33^. Surprisingly, a biopsy of a pleural-based nodule taken at this time revealed a poorly differentiated neuroendocrine tumor. DNA sequencing analysis of serial liquid biopsies collected over the previous 18 months provided evidence of an extremely rapid evolution of patient MP015’s cancer due to numerous progressive genetic amplifications, deletions, re-arrangements, and epigenetic changes. RNAseq analysis of lung metastases also demonstrated that a dramatic reduction in VGLL1 transcript expression (35.1 TPM to 1.6 TPM) had occurred between November 2013 and December 2015, providing a potential explanation for the lack of clinical response to ETC therapy (Supplementary Fig. 17). Patient MP015 expired in January 2016 due to extensive complications deriving from the progression of their lung metastases^33^.

### VGLL1-specific CTLs show cytotoxicity against PDAC tumor lines

Although patient MP015 did not experience clinical benefit from adoptive transfer of their own VGLL1-specific CTLs, the robust antitumor activity demonstrated by these T cells in vitro led us to explore whether they may have therapeutic potential for other PDAC patients. HLA-A*0101 was expressed by ~30% of our PDAC patient cohort, and RNAseq analysis of TCGA and MDACC PDAC surgical specimens and PDXs showed that 43.2–62.5% of patients express VGLL1 transcript at a level >5 TPM. From these data, it is estimated that 12–15% of PDAC patients present the LSELETPGKY peptide target in the context of HLA-A*0101 and therefore could potentially benefit from CTLs targeting VGLL1.

To determine if VGLL1 CTLs derived from patient MP015 could recognize allogeneic PDAC tumors, we tested a panel of HLA-A*0101-expressing PDAC tumor cell lines as targets for killing using a ^51^Cr release assay. Western blot analysis was used to confirm VGLL1 protein expression, and flow cytometry confirmed surface expression of HLA-A*0101 in cell lines (Supplementary Fig. 18). While control cell line WM793 (VGLL1-negative, HLA-A*0101-positive) was not recognized, VGLL1-specific CTLs recognized autologous MP015-Org cells and 4 out of 4 allogenic PDAC lines tested, including inducing robust killing of PANC10.05, CAPAN-1, and BXPC3 (Fig. 5a, b). The PDAC organoid cells derived from patient MP081 were also lysed by VGLL1 CTLs but with reduced efficiency, likely due to an outgrowth of VGLL1-negative cells within the culture. HLA class I specificity was demonstrated by co-incubation with the pan-MHC class I antibody W6/32, which resulted in blockade of PANC10.05 recognition and lysis (Supplementary Fig. 19). Collectively, these results provide evidence that the LSELETPGKY peptide constitutes a shared PDAC tumor antigen that can be effectively targeted with VGLL1-specific CTLs.

### VGLL1 CTLs show activity against multiple cancer cell lines

**Fig. 6:**
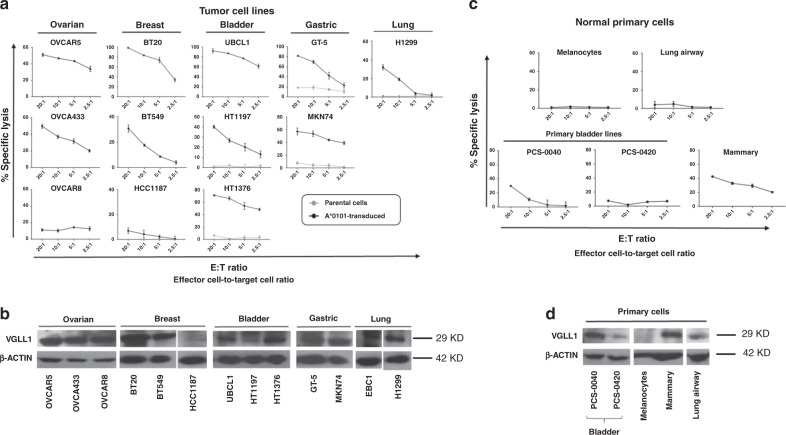
VGLL1-specific T cells recognize and kill multiple tumor types, but have reduced recognition of primary tissue cell lines. **a** VGLL1-specific CD8+ T cells were co-cultured with 12 different HLA-A*0101-expressing tumor cell lines derived from ovarian, lung, breast, bladder, or gastric cancer in a standard ^51^Cr release assay to measure cytotoxic activity at different effector-to-target (E:T) cell ratios. Five HLA-A*0101-negative cell lines (H1299, HT1197, HT1376, GT-5, and MKN74) were lentivirally transduced to stably express HLA-A*0101; specific killing of the parental cell lines (gray lines) are shown in comparison to HLA-A*0101-transduced counterparts (black lines). Data are presented as mean values from three independent experiments +/− SD. **b** Western blot analysis showing VGLL1 protein expression in tumor cell lines derived from ovarian, lung, breast, bladder, or gastric cancers was repeated three times with similar results. **c** VGLL1-specific CTLs were co-cultured with HLA-A*0101-expressing primary tissue cells derived from bladder, breast, lung airway, or skin melanocytes in a standard ^51^Cr release assay to measure cytotoxic activity. Cytolysis assay results show the means and standard deviations of six replicate samples, and data is representative of three independent experiments. **d** VGLL1 protein expression in primary cell lines as assessed by Western blot analysis was repeated three times with similar results.

TCGA RNAseq data analysis indicated that VGLL1 is expressed by several additional cancer types (16 of 34 total), most notably in 75–80% of patients with bladder, ovarian, and basal-type breast cancers, and 15–20% of patients with lung and gastric cancers (Fig. 3b). We therefore set out to determine whether cell lines derived from these cancer types could be targets for VGLL1-specific CTLs (Fig. 6a). Western blot analysis of a panel of ovarian, basal-type breast, bladder, gastric, and lung cancer cell lines showed high VGLL1 expression in 11 of 13 lines analyzed (Fig. 6b). Of the 8 cell lines that naturally expressed HLA-A*0101, VGLL1 CTLs killed 2 of 3 ovarian lines, 2 of 3 breast lines, and 1 of 1 bladder cancer lines (Fig. 6a). Interferon-gamma treatment of tumor cell lines did not significantly alter recognition and killing by VGLL1 CTLs (Supplementary Fig. 20). Five additional HLA-A*0101-negative cell lines (2 gastric, 2 bladder, and 1 lung line) were transduced to express HLA-A*0101 prior to testing them as targets for VGLL1 CTLs. As shown in Fig. 6a, all five HLA-A*0101-transduced cell lines were rendered susceptible to killing by VGLL1 CTLs, indicating presentation of the LSELETPGKY peptide from processed, endogenously-expressed VGLL1 protein. Taken together, these results suggest that VGLL1 CTLs have potential therapeutic value for at least five additional cancer types besides PDAC.

In order to assess the safety of VGLL1 CTLs for potential therapeutic use, we tested them against a panel of normal primary cells most likely to elicit VGLL1-specific reactivity according to the GTex normal tissue expression profile (Fig. 2a). Since bladder demonstrated the highest normal tissue *VGLL1* transcript expression, we tested two different HLA-A*0101-positive primary bladder cell lines as targets for killing. As shown in Fig. 6c, specific lysis was low, detectable in one bladder line but only at the highest E:T ratio. Since the GTex database indicated that *VGLL1* transcript is also expressed at low levels in normal breast and lung, we tested VGLL1-specific CTLs against HLA-A*0101-expressing primary mammary and lung airway cells, along with primary melanocytes as a negative control. Of this panel, mammary cells elicited moderately high levels of killing by VGLL1-specific CTLs, results that were consistent with VGLL1 protein levels as assessed by Western blot (Fig. 6d and Supplementary Fig. 22). By contrast, lung airway epithelial cells were not killed by VGLL1 CTLs, despite demonstrating ample HLA-A*0101 surface expression (Supplementary Fig. 18). These results provide evidence that VGLL1-specific T cells are unlikely to recognize essential normal tissues; however, safety concerns may be warranted due to the potential for reactivity against some non-essential tissues, including bladder and mammary tissue.

## Discussion

The benefits of immunotherapy have been slow to translate to PDAC patients, likely due to the relatively low mutational burden, highly suppressive tumor microenvironment, and a lack of known TAA targets for CTL therapies^32,45^. Oncogenic driver mutations in KRAS represent particularly promising target epitopes due to their tumor specificity and high prevalence in PDAC, colorectal cancer (CRC), and lung cancer. In an exciting recent case study, CTLs expanded from TILs of a CRC patient specifically recognized an HLA-C*0802-restricted KRAS peptide containing the G12D mutation; furthermore, these TILs were shown to mediate an objective tumor regression of multiple lung metastases in the patient following infusion^26^. While highly promising, the low worldwide prevalence of HLA-C*0802 predicts that only ~1.5% of PDAC patients could benefit from targeting this mutated epitope. TCRs recognizing mutated KRAS epitopes restricted to HLA-A*1101 have also been reported; although not yet tested in clinical trials, the relatively high prevalence of A*1101 predicts a significantly larger potential patient population that would be centered largely in Asia^46^. The lack of shared mutations beyond KRAS suggests that identification and targeting of non-mutated TAAs may represent the most promising opportunity for advancing immunotherapies for PDAC. Two well-studied TAAs for PDAC and ovarian cancer, MUC16, and CEACAM5, illustrate the two principal challenges of targeting non-mutated TAAs: difficulty in breaking T-cell tolerance and, conversely, the potential for induction of on-target off-tumor toxicities. Based on low overall normal tissue and relatively high tumor expression, MUC16 appears to be an ideal TAA; however, isolating high-affinity CTLs that recognize non-mutated MUC16 epitopes has proven elusive^23^^,^^47^. This lack of immunogenicity may be attributed to tolerogenic attributes of MUC16: being detectable at low levels in healthy patient serum, and also being a very large protein (>25,000 AA) containing >50 extracellular tandem repeat domains^48^. By contrast, *CEACAM5* transcript is highly expressed in tumors but also in normal colon and esophagus (>300 TPM), leading to severe colitis in patients treated with antigen-specific T cells^30^.

Employing an unbiased immunopeptidome analysis of tumor specimens derived from 35 PDAC patients, VGLL1 was identified as a putative shared TAA, ranked second only to MUC16 in terms of tumor expression in comparison to essential normal tissues. However, in contrast to MUC16 epitopes, the HLA-A*0101-restricted VGLL1 peptide was considerably more immunogenic, capable of eliciting antigen-specific CTLs from multiple donors, including one PDAC patient. Such immunogenicity provides a significant advantage in the context of developing endogenous T-cell (ETC) therapies for cancer patients. HLA-A*0101 is expressed at a relatively high prevalence (25–30%) in Western European and North American countries, suggesting that these patient populations would be most likely to benefit from targeting this epitope^49^. Expanded VGLL1-specific CTLs not only recognized and killed a panel of allogenic PDAC tumor lines, but also demonstrated reactivity against A*0101-expressing tumor cells derived from five other cancer types. We estimate that targeting this single VGLL1 epitope could potentially benefit a large number of Western cancer patients, including over 20% of patients with ovarian, bladder, or basal-like breast cancers, ~12% of patients with PDAC, and 5–8% of patients with lung, stomach, cervical, uterine, or head and neck cancers (Fig. 3).

Higher VGLL1 expression has been associated with shorter patient survival in multiple cancer types, including triple-negative breast and endometrial cancers^41,50^. However, its negative impact on survival is most striking in PDAC (Fig. 2b, c), suggesting that VGLL1 may play a role in driving tumor aggressiveness. VGLL1 is a co-transcriptional activator and a marker of proliferating cytotrophoblasts during early human placental development where it is co-expressed with the transcription factor TEAD4^43,51^. VGLL1 is thought to play a role in the Hippo signaling pathway, which controls organ size and is co-opted in multiple cancers to drive tumor progression^52,53^. Two well-characterized oncogenes, YAP and TAZ, function as co-transcriptional activators of the Hippo signaling pathway, and in cancers they also bind to TEAD proteins, leading to the upregulation of several cancer-promoting genes^39,54,55^. VGLL1 has been shown to interact with TEAD4 in a manner similar to that of YAP/TAZ, resulting in upregulation of the proliferation-promoting gene IGFBP5 and facilitating anchorage-independent cell proliferation^51^. These studies suggest that VGLL1 may promote cancer progression directly, which would increase its potential value as a therapeutic target. Although the *VGLL1* transcript loss observed in patient MP015 could argue against a role as an essential driver gene, the degree of tumor evolution documented in this patient’s cancer progression was exceptionally high^33^. Nonetheless, it does underscore the importance of antigen drift and the necessity of minimizing the time between personalized TAA identification and administration of TAA-specific CTL therapies. Further studies will be required to delineate the precise role of VGLL1 as a potential driver of tumorigenesis.

The discovery of VGLL1 prompted us to search for other putative CPAs that demonstrated overexpression in placenta and tumors, and thus may constitute potential TAA targets. This search uncovered placenta-specific 1 (PLAC1), initially identified as a target of autologous humoral immunity in gastric cancer and hepatocellular carcinoma patients, and the first CPA reported to represent a class of TAAs distinct from CTAs and oncofetal antigens^56,57^. A TCR recognizing an HLA-A*0201-restricted peptide derived from PLAC1 was recently isolated and shown to possess antitumor activity against human breast cancer cells in pre-clinical models, but have not yet been tested in clinical trials^58^. As shown in Fig. 3, *PLAC1* shows low normal tissue expression, but also demonstrates low overall prevalence in cancer. By contrast, insulin-like growth factor 2 mRNA binding protein 3 (IGF2BP3) was also identified as a promising CPA in our screen, showing relatively high prevalence of expression in ~15 different cancer types, including glioblastoma, uterine, testicular, and lung cancers (Fig. 3). The high level of *IGF2BP3* expression in normal testis, transformed lymphocytes and transformed fibroblasts suggest that this protein may also play a role in driving cancer progression, consistent with its identification as a poor prognostic factor^59,60^. Unfortunately, IGF2BP3 shows a significant degree of amino acid identity with IGF2BP2, which is expressed at elevated levels in several essential normal tissues, thus limiting the number of safely targetable epitopes. Of the 10 putative CPAs identified, matrix metallopeptidase 11 (*MMP11*) showed the most striking expression and prevalence, being expressed at high transcript levels in 25 different cancer types (Fig. 3). However, in addition to normal placenta, *MMP11* is also expressed at relatively high levels in uterus, cervix, and ovary, suggesting that CTL-based targeting of MMP11 epitopes may result in reproductive toxicities for women.

In terms of safety profile, cancer prevalence, and immunogenicity, VGLL1 compares favorably with other known TAA targets. Moreover, one PDAC patient treated with autologous, high-affinity VGLL1-specific CTLs experienced no apparent autoimmune toxicities, providing evidence that VGLL1 can be safely targeted in vivo. However, in vitro testing did demonstrate reactivity against cultured HLA-A*0101 primary mammary and bladder cells, suggesting that safety considerations should be taken into account when targeting CPAs. Immediate clinical applications of these findings include a planned clinical trial to treat HLA-A*0101^+^/VGLL1^+^ PDAC patients with VGLL1-specific ETC therapy, with future cohorts to potentially include bladder, ovarian, and/or breast cancer patients. Although an important limitation of the current study is the validation of VGLL1-specific CTL reactivity in only a single PDAC patient (MP015), VGLL1-specific TCRs derived from this patient have been cloned and are currently undergoing validation for future potential clinical applications, including TCR-T-cell therapies. MS-based identification of additional VGLL1 epitopes restricted to other HLA allotypes is also ongoing, with the promise of expanding the number of treatment-eligible cancer patients^61^. Although single antigen-based targeting can demonstrate limited clinical utility due to selection of antigen-loss variants, tumor debulking and subsequent epitope spreading constitute important aspects of immunotherapeutic success, processes that may be further augmented when combined with other modalities such as checkpoint blockade^15,16^. Collectively, our study shows that VGLL1 is a promising TAA target that can be used in immune-based therapies to address a serious unmet need in patients with PDAC and multiple other cancer types.

## Methods

### Cell lines

Human cancer cell lines demonstrating *VGLL1* mRNA expression were identified using the Cancer Cell Line Encyclopedia (CCLE) microarray-based gene expression analysis. HLA-A*0101-expressing cancer cell lines PANC10.05, CAPAN-1 OAW28, HT1197, HT1376, BXPC3, UBCL-1, and primary cell lines were obtained from commercial sources (ATCC and Sigma-Aldrich). The patient-derived organoid cell line MP015-Org (hMIA2D) was generated by the Tuveson lab at Cold Spring Harbor Labs^33^. The patient-derived organoid cell line MP081-Org was generated by the Maitra lab from tumor tissue derived from a wedge biopsy. The gastric cancer cell lines GT-5 and MKN74 were a kind gift from Dr. Lee Ellis. WM793, MKN74, PANC1005, GT-5, and OAW28 cells were cultured in RPMI 1640 medium (GIBCO), containing 10% fetal bovine serum, 1% penicillin–streptomycin (Pen-Strep) (Cellgrow), and 1% Insulin–Transferrin–Seleum-A (GIBCO). BT20 and bladder cell lines were cultured in equal parts DMEM F12K and MEM Alpha, with FBS, Pen-Strep, and 1% sodium pyruvate (GIBCO). All other cell lines were cultured in RPMI 1640, FBS, and 1% Pen-Strep, with the addition of HEPES (GIBCO) and Glutamax (GIBCO).

### Lentiviral transductions

Some HLA-A*0101-negative tumor cell lines that naturally expressed VGLL1 protein were transduced with a lentiviral gene transfer vector to express HLA-A*0101 driven by the human PGK promoter^62^. Ectopic cell surface expression of A*0101 was assessed by staining with anti-human HLA-A1-biotin and streptavidin-FITC (US Biological) and measuring fluorescence using a FACScanto II flow cytometer (BD Biosciences). Transduced tumor cells expressing physiological levels of surface HLA-A*0101 by fluorescence compared to tumor cells naturally expressing A*0101 were isolated by cell sorting using a FACSAria Fusion (BD Biosciences), expanded, and used in subsequent experiments.

### VGLL1 protein expression

VGLL1 protein expression was confirmed in all cell lines by western blot analysis. Cell lysates from tumor and primary cell lines were prepared and protein content normalized using the BCA method (Thermo-Fisher). Using standard Western blot techniques, cell lysates were run by polyacrylamide gel electrophoresis, transferred, and membranes probed with VGLL1-specific rabbit polyclonal antibody (TA322329, OriGene). VGLL1 protein was visualized using an enzyme-linked anti-rabbit mAb with the Scientific Pierce Fast Western Blot Kit, according to the manufacturer’s instructions. Full scan blots can be found in Supplementary Fig. 22.

### Mass spectrometry-based peptide identification

Patient-derived laparoscopic wedge biopsies, xenografts (PDX), or cell lines were lysed using Triton X-100 and cell lysates incubated overnight at 4 °C with 50 μg of pan-HLA-ABC specific mAb W6/32 for every 10 mg of protein. Protein A/G Ultralink resin beads were used to immunoprecipitate HLA class I molecules and HLA-bound peptides were then eluted with 0.1 M acetic acid. HLA-A,B,C isolation was confirmed by Western blot analysis, then HLA-positive elutes were analyzed by tandem mass spectrometry (MS/MS). HLA class I protein recovery was semi-quantitatively assessed by rating Western blot band intensity on a scale from 0 (not detectable) to 4 (highest intensity). Molecular weight filters were employed if tumor specimens were of sufficient size (>250 mg), but sample losses precluded this technique with smaller specimens. Eluted peptides were analyzed by high-sensitivity LC-MS/MS using a Nano-Cap LC system (Dionex Ultimate 3000 RSLC-Nano) interfaced to a tribrid mass spectrometer (Thermo Scientific Fusion) using ESI. Chromatography was performed using 0.1% formic acid in water as A solvent, 0.1% formic acid in acetonitrile as B solvent. Gradient initial conditions, 2% B for 5 min, gradient to 35% B over 40 min, 1 min ramp to 80% B, hold for 5 min, return to 2% B for re-equilibration. A 15 cm Column was packed with Phenomenex Kinetex 2.6 micron XB-C18, 100-angstrom particles. MS conditions electrospray source, full MS1 range was 265–1500 *m*/*z* at 240,000 resolution AGC settings, target ions 5-e5, maximum time 100 ms, for doubly charged precursors the selection range was limited to 400–750 *m*/*z*, for triply charged precursors the selection range was limited to 270–500 *m*/*z*. MS/MS was performed in the linear ion trap, AGC settings, target ions 4-e5, maximum time, 300 ms, isolation window 0.7 da, cycle time between master scans, 1.5 s, number of precursors, automatic, dynamic exclusion time 20 s. To analyze the acquired MS/MS spectra, the Mascot algorithm was utilized to search the spectra against the SwissProt complete human protein database (updated 9/2018), which provided potential matches to conventionally annotated peptides. In selected cases, targeted-MS/MS analysis was performed to confirm TAA peptide identity. For these analyses, retention-time windows for ^13^C/^15^N isotope-labeled synthetic peptide standards were pre-determined by MS analysis of the synthetic peptides, then targeted methods for searching TAA peptides were constructed using mass windows of 3 Da around each *m*/*z*. Peptide spectra are shown with *y*^+^ fragment ions color-coded blue, *b*^+^ fragment ions red, and non-matching fragments in gray. The mass spectrometry proteomics data have been deposited to the ProteomeXchange Consortium via the PRIDE partner repository with the dataset identifier PXD018302 (https://www.ebi.ac.uk/pride/).

### Peptide selection and validation

Individual peptide matches underwent quality assessment by reference to multiple orthogonal parameters, including Mascot Ion score, MS1 measured differential to the calculated peptide mass (delta mass), and predicted binding to the patient’s HLA allotypes as determined by high-resolution genetic sequencing and the NetMHC and NetMHCpan algorithms^34,35^. Peptide matches were analyzed by BLAST searches to identify all potential source genes, which were then cross-referenced to RNAseq data derived from individual tumor samples to provide further validation of peptide identity (validation requiring a minimum source gene expression of 0.3 transcripts per million, TPM). Eluted TAA peptides were screened for safety as potential CTL targets by applying sequential RNA transcript expression filters to eliminate peptides most likely to elicit autoimmune toxicities due to normal tissue expression (GTex Portal RNAseq data, http://cancergenome.nih.gov/). Excluding testis and placenta, source gene transcript expression of 30 TPM maximum was allowed in non-essential tissues (listed in Supplementary Table 2), 10 TPM in caution tissues, 3 TPM in hazard tissues, and 1 TPM in highly essential danger tissues (such as heart and brain). Putative TAA genes were also screened for expression and prevalence in different cancer types through analysis of TCGA RNAseq data (http://cancergenome.nih.gov/). The RNAseq TPM thresholds were chosen based on the following reasons: Two published studies that have reported fatalities from adoptive transfer of engineered antigen-specific TCR-T cells have shown that MAGEA3-specific T cells cross-reacted with either a Titin-derived peptide in the heart (where GTex Titin expression is ~60 TPM), causing cardiac arrest; or cross-reacted with a MAGEA12 peptide in the brain (where the GTex database showed MAGEA12 expression was median ~0.3 TPM)^37,63^. We chose 1 TPM as the safety threshold for TAAs expressed by the heart and brain, which were considered danger tissues; however, it is possible that antigens expressed at levels lower than 1 TPM may be recognized by T cells. Lung, liver, colon, stomach, and esophagus were considered as hazard tissues since though they are clearly essential, T-cell therapies have not been shown to lead to any fatal cross-reactivities. We therefore chose a threshold 3-fold higher (3 TPM) as an acceptable level of expression for these tissues. We chose 10 TPM as threshold for hematopoietic tissues and 30 TPM for non-essential tissues, since targeting of these tissues with tumor-antigen-specific T cells would not be likely to lead to fatalities, although we cannot rule out autoimmune toxicities, as we discuss in the manuscript. In our experience attempting to derive and expand cytotoxic T cells against multiple non-mutated peptides from donor peripheral blood, we have found that it is highly challenging to generate T cells against genes with expression >30 TPM in any normal tissue (likely due to deletional tolerance), which determined the upper TPM limit.

### Gene expression analysis and patient survival

Whole transcriptome sequencing (RNAseq) analysis was performed on RNA derived from all PDAC tumor specimens, xenografts, and organoid cell lines using the Illumina TruSeq Stranded Total RNA kit with Ribo-Zero Gold with ~200 million paired-end reads for each tumor RNA sample (Avera Institute for Human Genetics). Gene expression profiles of *VGLL1* and other cancer-placenta antigens were determined by compiling RNAseq data derived from normal human primary tissues (GTex Portal) and tumor tissues (TCGA). Kaplan–Meier curves were generated from survival data of TCGA cancer patients when stratified by tumor *VGLL1* transcript expression.

### Isolation and expansion of VGLL1-specific CD8 T cells

Tumor-antigen-specific CTLs were generated as previously described^64–66^. HLA-A*0101-positive patient- or healthy donor-derived PBMCs were stimulated twice by autologous dendritic cells (DCs) pulsed with the VGLL1_231-240_ peptide LSELETPGKY. Six days after the second DC stimulation, cultured cells were stained with VGLL1_231-240_ peptide/HLA-A*0101–PE-conjugated custom tetramer (1:150 dilution, Fred Hutchinson Cancer Research Center), washed, and then stained with APC-conjugated CD8 antibody (1:25 dilution). Cells were washed and analyzed by flow cytometry (LSRFortessa X-20 Analyzer). CD8 and tetramer double-positive cells were sorted by ARIA II and the VGLL1-specific CD8 T cells were expanded using the Rapid Expansion Protocol (REP) with PBMCs and EBV-immortalized lymphoblastoid B-cell lines (LCL) feeder cells. The TCR-V_β_ repertoire of expanded CD8 T cells was assessed using the IOTest Beta Mark TCR-V_β_ Repertoire kit. An example of the CTL gating strategy can be found in Supplementary Fig. 21.

### Cytotoxic T-cell assays

Antitumor killing by VGLL1-specific CD8+ T cells was assessed using a standard chromium-51 (^51^Cr) release assay. Target cells were labeled with 100 μL of ^51^Cr for 1 h, then washed and plated at 2000 target cells per well in triplicate. VGLL1-specific CD8+ T cells were incubated with target cells at various effector-to-target (E:T) cell ratios for four hours. After the incubation period, supernatant was collected from the wells and ^51^Cr was measured with a gamma radiation counter. The percentage of specific target cell lysis was calculated, correcting for background ^51^Cr release and relative to a maximum ^51^Cr release as measured by Triton X-100 lysed target cells.

### Adoptive transfer of VGLL1-specific T cells

All clinical investigations were conducted according to the Declaration of Helsinki principles. PDAC patient MP015 presented with metastatic pancreatic cancer and underwent several lines of conventional and experimental therapy over a 4-year period including FOLFIRINOX, Bevacizumab, GVAX vaccine, and Gemcitabine/Abraxane that ultimately failed to control the disease. Since this patient was refractory to treatment and had exhausted conventional therapeutic options, it was deemed appropriate to consent and treat them under a Compassionate Use Investigational Drug (CIND) protocol approved by the U.S. Food and Drug Administration (FDA) and the M.D. Anderson Cancer Center clinical trials IRB committee^33^. The generation of antigen-specific CTL was performed according to methods established in the Yee Lab^15^, summarized briefly as follows: PBMCs were collected by leukapheresis and all ensuing ex vivo manipulations were performed in the Cell Processing Facility at UT MD Anderson Cancer Center. Donor PBMC was stimulated two times 7 days apart, with autologous dendritic cells (DC) pulsed with the VGLL1 peptide in the presence of IL-21 (30 ng ml^−1^). Previous studies have shown that exposure of T cells to IL-21 during in vitro priming leads to the generation of a unique population of central memory type CD8 T cells (TCM) characterized by high surface expression of CD28 and CD127 (typically 40–90% CD28^+^CD127^+^) that enables long-term in vivo persistence of transferred T cells^16,66,67^. On Day 2 of each stimulation, IL-2 (12.5 IU ml^−1^), IL-7 (5 ng ml^−1^), and IL-15 (1 ng ml^−1^) were added to obtain sufficient frequencies (>1% by multimer staining) of VGLL1-reactive CD8+ T cells which were then selected by clinical-grade multimer-guided sorting (Nanosorter, Owl Biomedical) and expanded using the Rapid Expansion Protocol to >1e10 cells. Under the protocol, the patient received very low-dose cyclophosphamide (300 mg m^−2^ iv) conditioning on Day −2 and then an infusion of 1.9e10 polyclonal, IL-21 primed antigen-specific CTL m^−2^, immediately followed by a 2-week course of low-dose s.c. IL-2 and PD1 blockade (3 mg kg^−1^ every 2 weeks × 16 doses). Radiologic responses were evaluated according to the mWHO-based irRC Criteria^33^. Although the patient was hospitalized for monitoring of potential cell-infusion-associated adverse events (AEs), no serious AEs were observed apart from expected transient (<24 h) culture-negative fevers (≥38.3 °C), associated with CTL-induced cytokine release syndrome, and lymphopenia lasting <10 days^33^.

### Statistical analysis

Data analysis was performed using GraphPad Prism version 7.03. Normally distributed data were analyzed using parametric tests (ANOVA or unpaired *t* test). Kaplan–Meier survival curves were analyzed by log-rank tests. Test differences were considered statistically significant if *P* < 0.05.

### Reporting summary

Further information on research design is available in the Nature Research Reporting Summary linked to this article.

## Supplementary information

Supplementary Information

Description of Additional Supplementary Files

Supplementary Dataset 1

Supplementary Dataset 2

Reporting Summary

## Data Availability

The raw mass spectrometry data of peptides eluted from organoid lines MP015 and MP081 have been deposited in the ProteomeXchange Consortium database via the PRIDE partner repository under the dataset identifier PXD018302 (https://www.ebi.ac.uk/pride/). Complete MS search results and lists of peptides eluted from PDAC patients MP015 and MP081 are shown in Supplementary Data 1 and 2. The GTex Portal database and TCGA data referenced during the study are available in public repositories from websites http://www.gtexportal.org/home/ and http://cancergenome.nih.gov/. All other data supporting the findings of this study are available within the article and its supplementary information files, or from the corresponding authors upon reasonable request. Correspondence to Cassian Yee (cyee@mdanderson.org) or Gregory Lizee (glizee@mdanderson.org). Complete raw patient RNAseq data has been deposited in the European Nucleotide Archive under the study accession number PRJEB40462 (https://www.ebi.ac.uk/ena/browser/home). A reporting summary for this article is available as a Supplementary Information file.

## References

[1] Varghese AM, Lowery MA, Yu KH, O’Reilly EM (2016). Current management and future directions in metastatic pancreatic adenocarcinoma. Cancer.

[2] McGuigan A (2018). Pancreatic cancer: a review of clinical diagnosis, epidemiology, treatment and outcomes. World J. Gastroenterol..

[3] Chari ST (2015). Early detection of sporadic pancreatic cancer: summative review. Pancreas.

[4] Polireddy K, Chen Q (2016). Cancer of the pancreas: molecular pathways and current advancement in treatment. J. Cancer.

[5] Van Allen EM (2015). Genomic correlates of response to CTLA-4 blockade in metastatic melanoma. Science.

[6] Strobel O, Neoptolemos J, Jager D, Buchler MW (2019). Optimizing the outcomes of pancreatic cancer surgery. Nat. Rev. Clin. Oncol..

[7] Lizee G (2013). Harnessing the power of the immune system to target cancer. Annu Rev. Med.

[8] Wei SC, Duffy CR, Allison JP (2018). Fundamental mechanisms of immune checkpoint blockade therapy. Cancer Disco..

[9] Rizvi NA (2015). Mutational landscape determines sensitivity to PD-1 blockade in non-small cell lung cancer. Science.

[10] Dudley ME (2005). Adoptive cell transfer therapy following non-myeloablative but lymphodepleting chemotherapy for the treatment of patients with refractory metastatic melanoma. J. Clin. Oncol..

[11] Radvanyi LG (2012). Specific lymphocyte subsets predict response to adoptive cell therapy using expanded autologous tumor-infiltrating lymphocytes in metastatic melanoma patients. Clin. Cancer Res..

[12] Tran E (2015). Immunogenicity of somatic mutations in human gastrointestinal cancers. Science.

[13] Zacharakis N (2018). Immune recognition of somatic mutations leading to complete durable regression in metastatic breast cancer. Nat. Med..

[14] Hinrichs CS, Rosenberg SA (2014). Exploiting the curative potential of adoptive T-cell therapy for cancer. Immunol. Rev..

[15] Chapuis AG (2016). T-cell therapy using interleukin-21-primed cytotoxic T-cell lymphocytes combined with cytotoxic T-cell lymphocyte antigen-4 blockade results in long-term cell persistence and durable tumor regression. J. Clin. Oncol..

[16] Chapuis AG (2016). Combined IL-21-primed polyclonal CTL plus CTLA4 blockade controls refractory metastatic melanoma in a patient. J. Exp. Med..

[17] Robbins PF (2015). A pilot trial using lymphocytes genetically engineered with an NY-ESO-1-reactive T-cell receptor: long-term follow-up and correlates with response. Clin. Cancer Res..

[18] Tawara I (2017). Safety and persistence of WT1-specific T-cell receptor gene-transduced lymphocytes in patients with AML and MDS. Blood.

[19] Morgan RA (2006). Cancer regression in patients after transfer of genetically engineered lymphocytes. Science.

[20] Young K, Hughes DJ, Cunningham D, Starling N (2018). Immunotherapy and pancreatic cancer: unique challenges and potential opportunities. Ther. Adv. Med. Oncol..

[21] Kabacaoglu D, Ciecielski KJ, Ruess DA, Algul H (2018). Immune checkpoint inhibition for pancreatic ductal adenocarcinoma: current limitations and future options. Front Immunol..

[22] Yarchoan M, Hopkins A, Jaffee EM (2017). Tumor mutational burden and response rate to PD-1 inhibition. N. Engl. J. Med..

[23] Balachandran VP (2017). Identification of unique neoantigen qualities in long-term survivors of pancreatic cancer. Nature.

[24] Bailey P (2016). Genomic analyses identify molecular subtypes of pancreatic cancer. Nature.

[25] Foucher ED (2018). Pancreatic ductal adenocarcinoma: a strong imbalance of good and bad immunological cops in the tumor microenvironment. Front Immunol..

[26] Tran E (2016). T-Cell Transfer Therapy Targeting Mutant KRAS in Cancer. N. Engl. J. Med..

[27] Beatty GL (2018). Activity of mesothelin-specific chimeric antigen receptor T cells against pancreatic carcinoma metastases in a phase 1 trial. Gastroenterology.

[28] Stromnes IM (2015). T cells engineered against a native antigen can surmount immunologic and physical barriers to treat pancreatic ductal adenocarcinoma. Cancer Cell.

[29] Koneru M, O’Cearbhaill R, Pendharkar S, Spriggs DR, Brentjens RJ (2015). A phase I clinical trial of adoptive T cell therapy using IL-12 secreting MUC-16(ecto) directed chimeric antigen receptors for recurrent ovarian cancer. J. Transl. Med..

[30] Parkhurst MR (2011). T cells targeting carcinoembryonic antigen can mediate regression of metastatic colorectal cancer but induce severe transient colitis. Mol. Ther..

[31] Akce M, Zaidi MY, Waller EK, El-Rayes BF, Lesinski GB (2018). The potential of CAR T cell therapy in pancreatic cancer. Front Immunol..

[32] Morrison AH, Byrne KT, Vonderheide RH (2018). Immunotherapy and Prevention of Pancreatic Cancer. Trends Cancer.

[33] Wolff RA (2018). Dynamic changes during the treatment of pancreatic cancer. Oncotarget.

[34] Jurtz V (2017). NetMHCpan-4.0: improved peptide-MHC class I interaction predictions integrating eluted ligand and peptide binding affinity data. J. Immunol..

[35] Nielsen M, Andreatta M (2016). NetMHCpan-3.0; improved prediction of binding to MHC class I molecules integrating information from multiple receptor and peptide length datasets. Genome Med..

[36] Linette GP (2013). Cardiovascular toxicity and titin cross-reactivity of affinity-enhanced T cells in myeloma and melanoma. Blood.

[37] Morgan RA (2013). Cancer regression and neurological toxicity following anti-MAGE-A3 TCR gene therapy. J. Immunother..

[38] Schuster H (2017). The immunopeptidomic landscape of ovarian carcinomas. Proc. Natl Acad. Sci. USA.

[39] Vaudin P, Delanoue R, Davidson I, Silber J, Zider A (1999). TONDU (TDU), a novel human protein related to the product of vestigial (vg) gene of Drosophila melanogaster interacts with vertebrate TEF factors and substitutes for Vg function in wing formation. Development.

[40] Simon E, Faucheux C, Zider A, Theze N, Thiebaud P (2016). From vestigial to vestigial-like: the Drosophila gene that has taken wing. Dev. Genes Evol..

[41] Castilla MA (2014). VGLL1 expression is associated with a triple-negative basal-like phenotype in breast cancer. Endocr. Relat. Cancer.

[42] Cheng L (2011). Sarcomatoid carcinoma of the urinary bladder: the final common pathway of urothelial carcinoma dedifferentiation. Am. J. Surg. Pathol..

[43] Soncin, F. et al. Comparative analysis of mouse and human placentae across gestation reveals species-specific regulators of placental development. *Development* **145**, 10.1242/dev.156273 (2018). 10.1242/dev.156273 PMC5825847 29361559

[44] Dong XY (2008). Plac1 is a tumor-specific antigen capable of eliciting spontaneous antibody responses in human cancer patients. Int J. Cancer.

[45] Torphy RJ, Zhu Y, Schulick RD (2018). Immunotherapy for pancreatic cancer: barriers and breakthroughs. Ann. Gastroenterol. Surg..

[46] Wang QJ (2016). Identification of T-cell receptors targeting KRAS-mutated human tumors. Cancer Immunol. Res.

[47] Bellone S (2009). Generation of CA125-specific cytotoxic T lymphocytes in human leukocyte antigen-A2.1-positive healthy donors and patients with advanced ovarian cancer. Am. J. Obstet. Gynecol..

[48] Aithal A (2018). MUC16 as a novel target for cancer therapy. Expert Opin. Ther. Targets.

[49] Gonzalez-Galarza FF (2015). Allele frequency net 2015 update: new features for HLA epitopes, KIR and disease and HLA adverse drug reaction associations. Nucleic Acids Res..

[50] Uhlen M (2015). Tissue-based map of the human proteome. Science.

[51] Pobbati AV, Chan SW, Lee I, Song H, Hong W (2012). Structural and functional similarity between the Vgll1-TEAD and the YAP-TEAD complexes. Structure.

[52] Mesrouze Y (2014). The surprising features of the TEAD4-Vgll1 protein-protein interaction. Chembiochem.

[53] Moya IM, Halder G (2018). Hippo-YAP/TAZ signalling in organ regeneration and regenerative medicine. Nat. Rev. Mol. Cell Biol..

[54] Zhang X (2018). The role of YAP/TAZ activity in cancer metabolic reprogramming. Mol. Cancer.

[55] Zhou, Y. et al. The TEAD family and its oncogenic role in promoting tumorigenesis. *Int. J. Mol. Sci.* **17**, 10.3390/ijms17010138 (2016). 10.3390/ijms17010138 PMC4730377 26805820

[56] Chen J (2006). PLAC1/CP1 gene expression and autologous humoral immunity in gastric cancer patients. Beijing Da Xue Xue Bao Yi Xue Ban..

[57] Silva WA (2007). PLAC1, a trophoblast-specific cell surface protein, is expressed in a range of human tumors and elicits spontaneous antibody responses. Cancer Immun..

[58] Li Q (2018). PLAC1-specific TCR-engineered T cells mediate antigen-specific antitumor effects in breast cancer. Oncol. Lett..

[59] Shi R (2017). Expression profile, clinical significance, and biological function of insulin-like growth factor 2 messenger RNA-binding proteins in non-small cell lung cancer. Tumour Biol..

[60] Zhou Y (2017). IGF2BP3 functions as a potential oncogene and is a crucial target of miR-34a in gastric carcinogenesis. Mol. Cancer.

[61] Park J (2017). SLC45A2: a melanoma antigen with high tumor selectivity and reduced potential for autoimmune toxicity. Cancer Immunol. Res..

[62] Bradley SD (2015). BRAFV600E co-opts a conserved MHC class I internalization pathway to diminish antigen presentation and CD8+ T-cell recognition of melanoma. Cancer Immunol. Res..

[63] Cameron BJ (2013). Identification of a Titin-derived HLA-A1-presented peptide as a cross-reactive target for engineered MAGE A3-directed T cells. Sci. Transl. Med..

[64] Li Y, Bleakley M, Yee C (2005). IL-21 influences the frequency, phenotype, and affinity of the antigen-specific CD8 T cell response. J. Immunol..

[65] Li Y, Yee C (2008). IL-21 mediated Foxp3 suppression leads to enhanced generation of antigen-specific CD8+ cytotoxic T lymphocytes. Blood.

[66] Chapuis AG (2013). Transferred WT1-reactive CD8+ T cells can mediate antileukemic activity and persist in post-transplant patients. Sci. Transl. Med..

[67] Wang J (2020). Histone deacetylase inhibitors and IL21 cooperate to reprogram human effector CD8(+) T cells to memory T cells. Cancer Immunol. Res..

