# Supplementary material for: Vestigial-like 1 is a shared targetable cancer-placenta antigen expressed by pancreatic and basal-like breast cancers
Source: Nat Commun. 2020 Oct 21;11:5332. doi: 10.1038/s41467-020-19141-w (PMC7577998; doi:10.1038/s41467-020-19141-w)
Supplement: Supplementary file 1 — Supplementary Information [file 41467_2020_19141_MOESM1_ESM.pdf]

VESTIGIAL-LIKE 1 IS A SHARED TARGETABLE CANCER-PLACENTA ANTIGEN EXPRESSED BY PANCREATIC AND BASAL-LIKE BREAST CANCER

Bradley et al.

Supplementary Figure 1.
VGLL1-derived peptide eluted from PDAC cell line PANC10.05

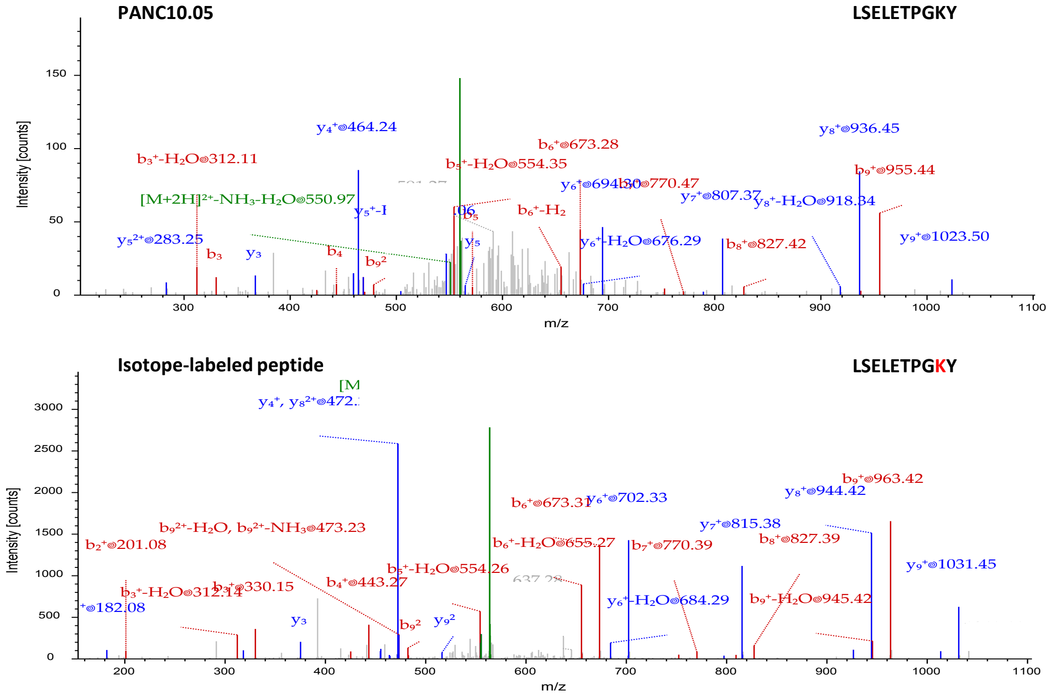

Supplementary Figure 1. VGLL1-derived peptide was eluted from the PANC-1005 cell line. Mass spectra of HLA-A*0101-restricted VGLL1-derived peptide isolated from PDAC cell line PANC10.05 (top panel). The native peptide co-eluted with and matched the MS fragmentation spectra of the synthetic isotope-labeled peptide LSELETPGKY containing a $^{13}C/^{15}N$-labeled lysine residue (bottom panel).

Supplementary Figure 2. Basal-like subset of Breast Cancer shows elevated VGLL1 Expression

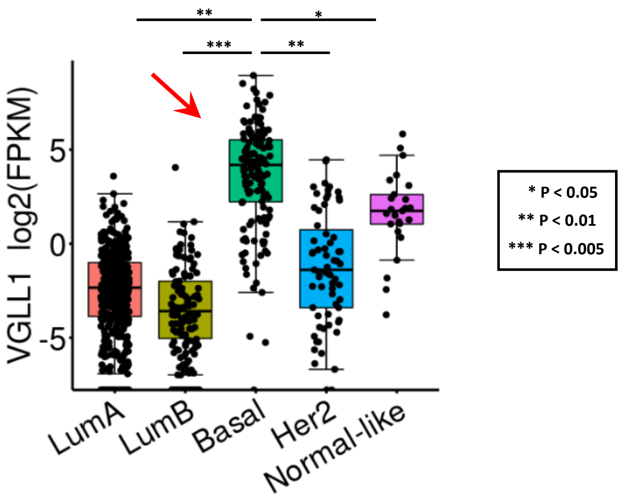

The Cancer Genome Atlas (TCGA)

Supplementary Figure 2. VGLL1 is preferentially expressed in basal-like breast cancer compared to other breast cancer subtypes. TCGA breast cancer patients were subdivided into 5 major sub-types (LumA, LumB, Basal-like, HER2 overexpressing, and normal-like) and analyzed for tumor VGLL1 expression by RNAseq analysis. Each dot represents one TCGA patient sample, and VGLL1 transcript expression is expressed in fragments per kilobase of transcript per million mapped reads (FPKM). Data are represented as center (mean) with bounds of the box containing the 50th percentile while the whiskers contain minima (25th percentile) and maxima (75th percentile).

Supplementary Figure 3. VGLL1 prevalence and expression in CCLE tumor cell lines

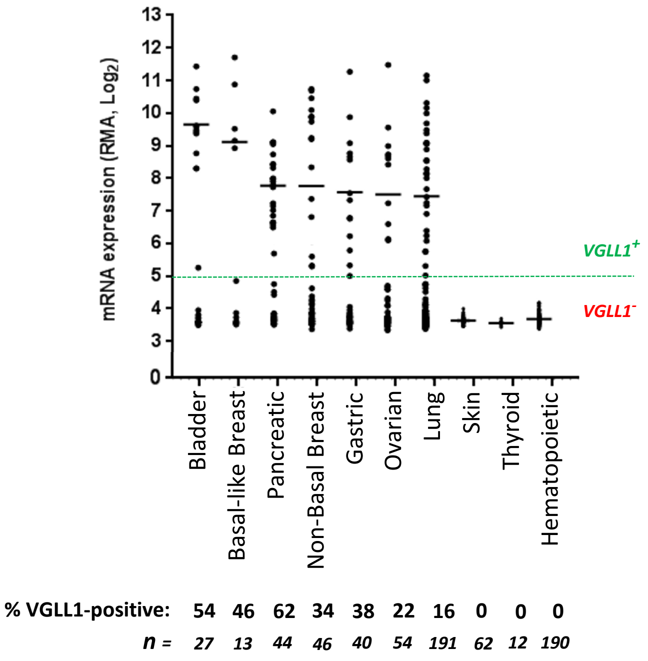

Supplementary Figure 3. VGLL1 gene expression in tumor cell lines derived from a variety of cancer types. Gene expression microarray analysis of a diverse array of tumor cell lines (n=679) from the Cancer Cell Line Encyclopedia (CCLE) showed that VGLL1 is expressed by a majority of PDAC and bladder cancer cell lines, in addition to a significant percentage of breast, gastric, ovarian, and lung cancer cell lines. No VGLL1 expression was found in cell lines derived from skin, thyroid, or hematopoietic cancers. Threshold for VGLL1 antigen positivity was 3-fold above background signal.

Supplementary Figure 4. High tumor VGLL1 expression is associated with reduced survival in stomach and breast cancers

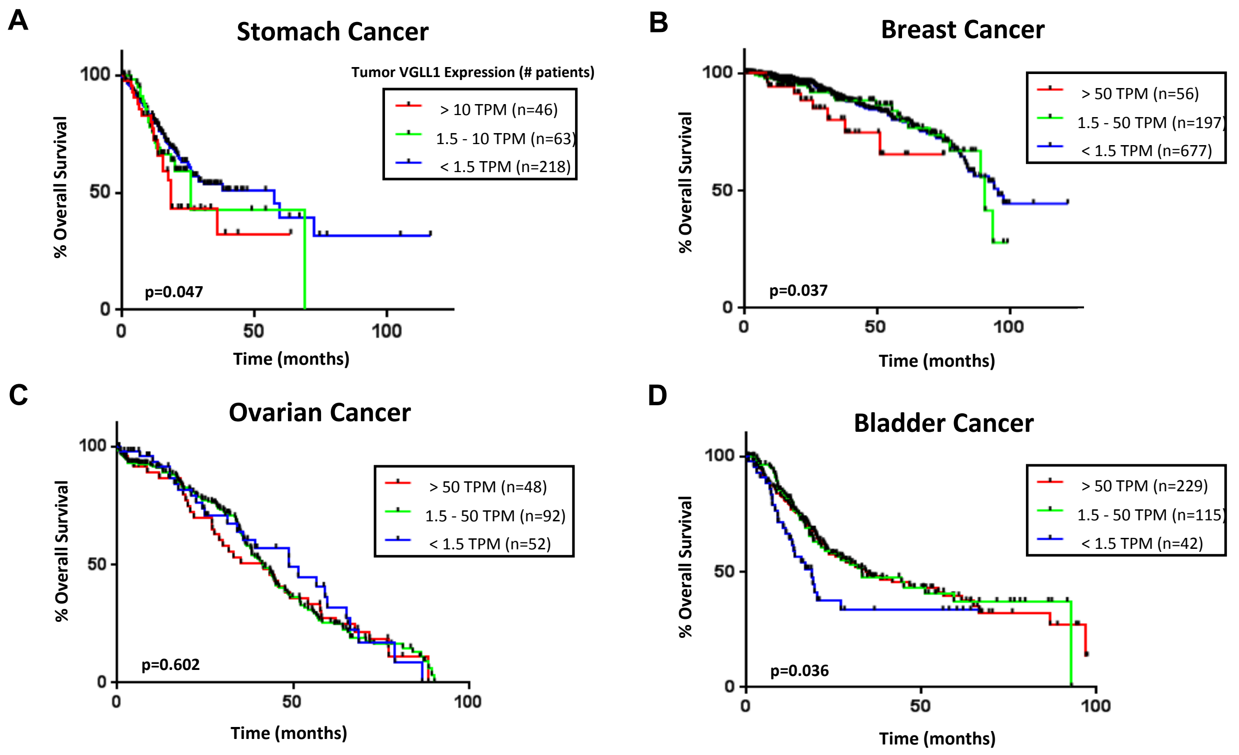

Supplementary Figure 4. High tumor VGLL1 expression is associated with reduced survival in multiple cancer types. TCGA cancer patients were stratified into three groups according to tumor VGLL1 expression as determined by RNAseq analysis. Kaplan-Meier curves show overall survival (OS) of each group for **(A)** Stomach adenocarcinoma, **(B)** Breast carcinoma, **(C)** Ovarian serous adenocarcinoma, and **(D)** bladder urothelial carcinoma patients. P-values indicate log-rank significance test results comparing the survival curve differences using Gehan-Breslow-Wilcoxon test.

Supplementary Figure 5. Mean expression of cancer-placenta antigens in all TCGA tumor specimens

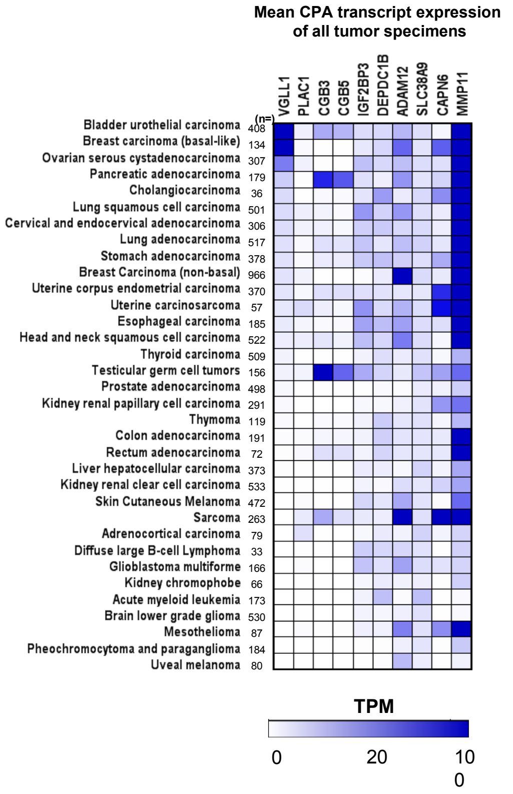

Supplementary Figure 5. Expression of cancer-placenta antigens (CPAs) in all TCGA tumor specimens. Gene expression profiling to search for potential TAAs with similar expression profiles to VGLL1 uncovered nine additional putative CPAs. Heat map depicts the mean transcript expression of all 10 CPAs in 34 different TCGA cancer types, as determined by RNAseq. Tumor tissues are listed in order of highest to lowest mean VGLL1 transcript expression.

Supplementary Figure 6
GTex / TCGA expression profile – VGLL1

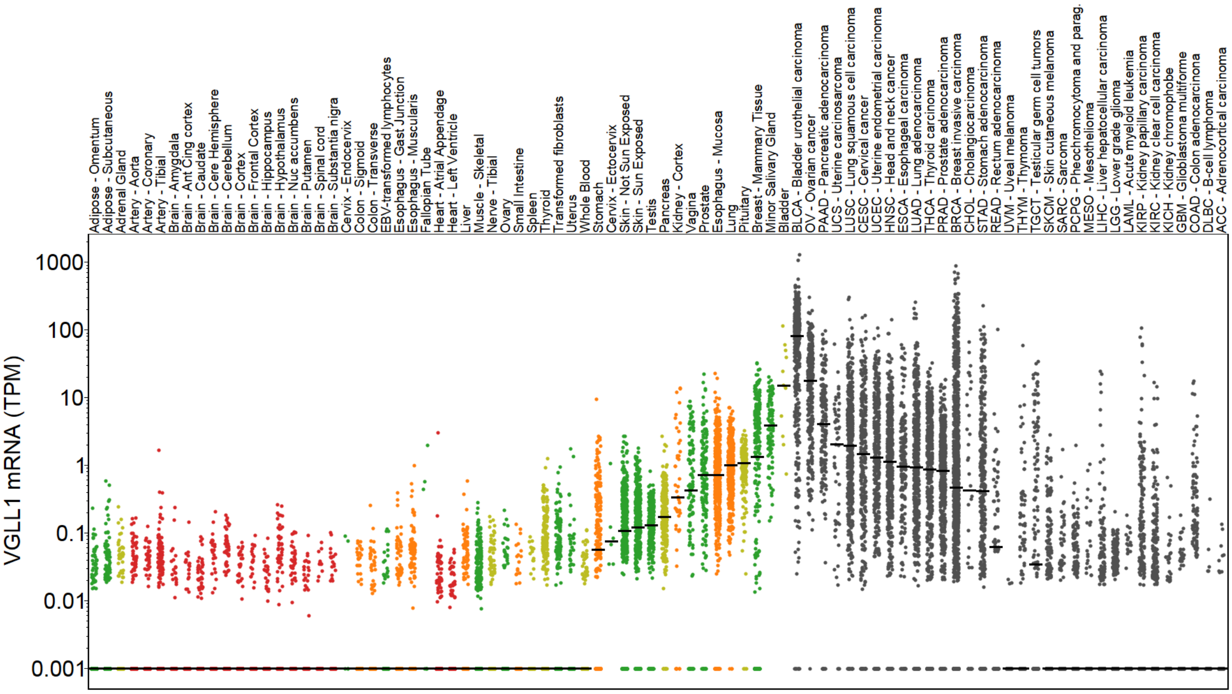

Supplementary Figure 6. Gene expression profile of VGLL1. Transcript expression of VGLL1 in normal tissues (colored dots, GTex Portal database) and human cancers (black dots, TCGA database), as determined by RNAseq analyses. Each dot represents one normal donor or patient tumor sample. Colors correspond to the four normal tissue categories defined in Figure 1B and Supplemental Table 2: Green, non-essential tissues; Yellow, caution tissues; Orange, hazard tissues; Red, danger tissues.

Supplementary Figure 7
GTex / TCGA expression profile – PLAC1

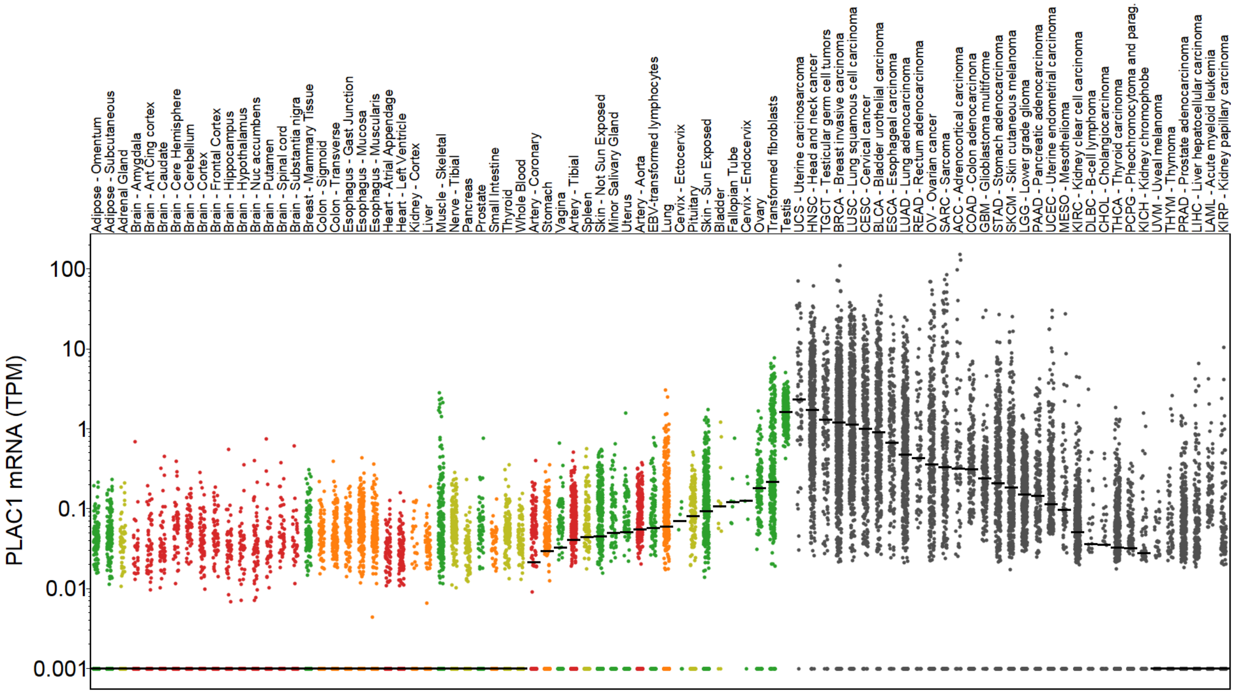

Supplementary Figure 7. Gene expression profile of PLAC1. Transcript expression of PLAC1 in normal tissues (colored dots, GTex Portal database) and human cancers (black dots, TCGA database), as determined by RNAseq analyses. Each dot represents one normal donor or patient tumor sample. Colors correspond to the four normal tissue categories defined in Figure 1B and Supplemental Table 2: Green, non-essential tissues; Yellow, caution tissues; Orange, hazard tissues; Red, danger tissues.

Supplementary Figure 8
GTex / TCGA expression profile – CGB3

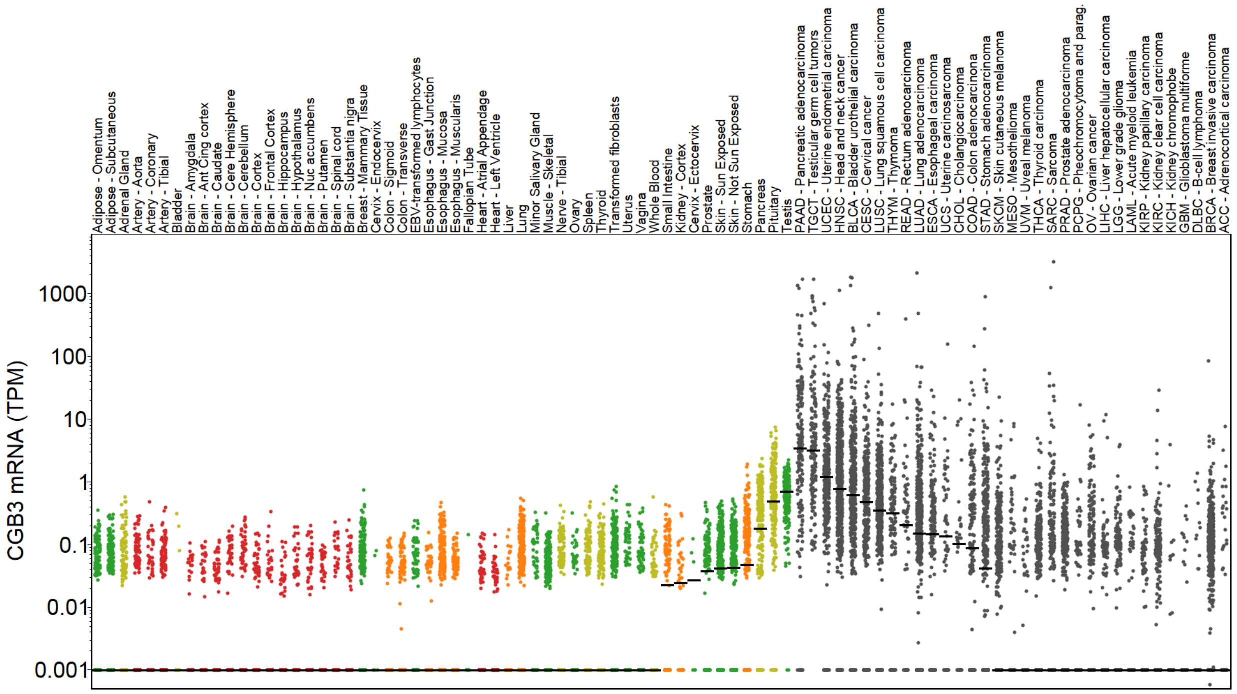

Supplementary Figure 8. Gene expression profile of CGB3. Transcript expression of CGB3 in normal tissues (colored dots, GTex Portal database) and human cancers (black dots, TCGA database), as determined by RNAseq analyses. Each dot represents one normal donor or patient tumor sample. Colors correspond to the four normal tissue categories defined in Figure 1B and Supplemental Table 2: Green, non-essential tissues; Yellow, caution tissues; Orange, hazard tissues; Red, danger tissues.

Supplementary Figure 9
GTex / TCGA expression profile – CGB5

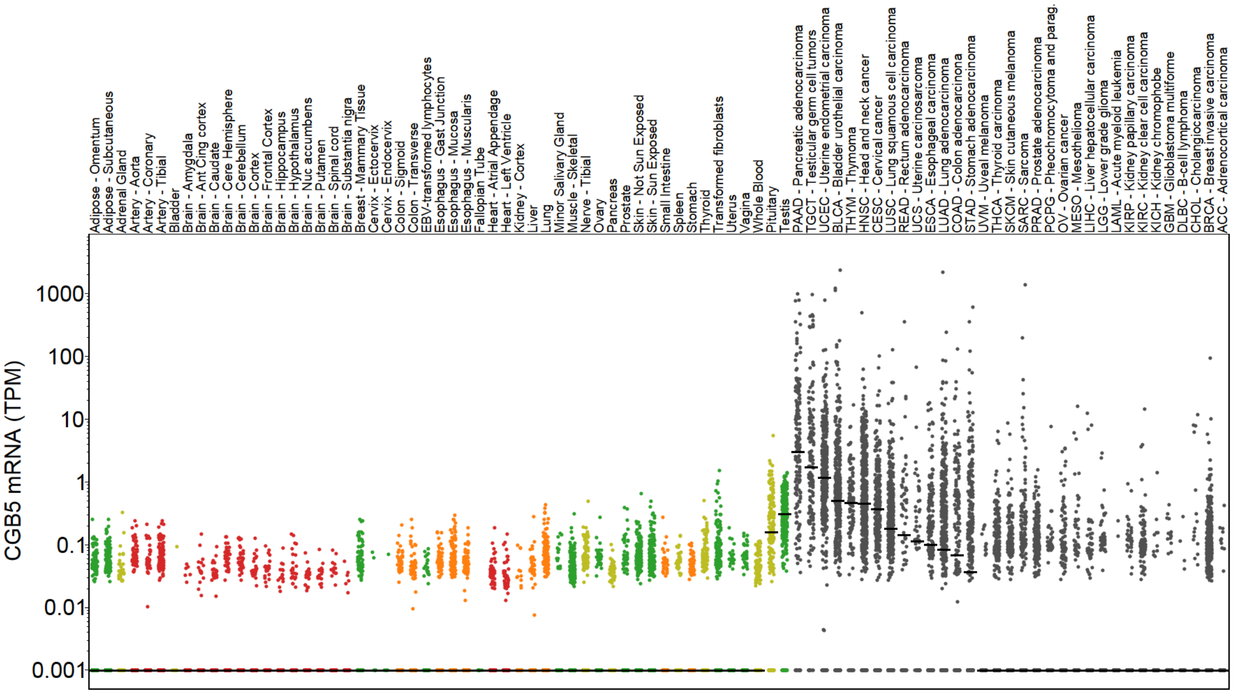

Supplementary Figure 9. Gene expression profile of CGB5. Transcript expression of CGB5 in normal tissues (colored dots, GTex Portal database) and human cancers (black dots, TCGA database), as determined by RNAseq analyses. Each dot represents one normal donor or patient tumor sample. Colors correspond to the four normal tissue categories defined in Figure 1B and Supplemental Table 2: Green, non-essential tissues; Yellow, caution tissues; Orange, hazard tissues; Red, danger tissues.

Supplementary Figure 10
GTex / TCGA expression profile – IGF2BP3

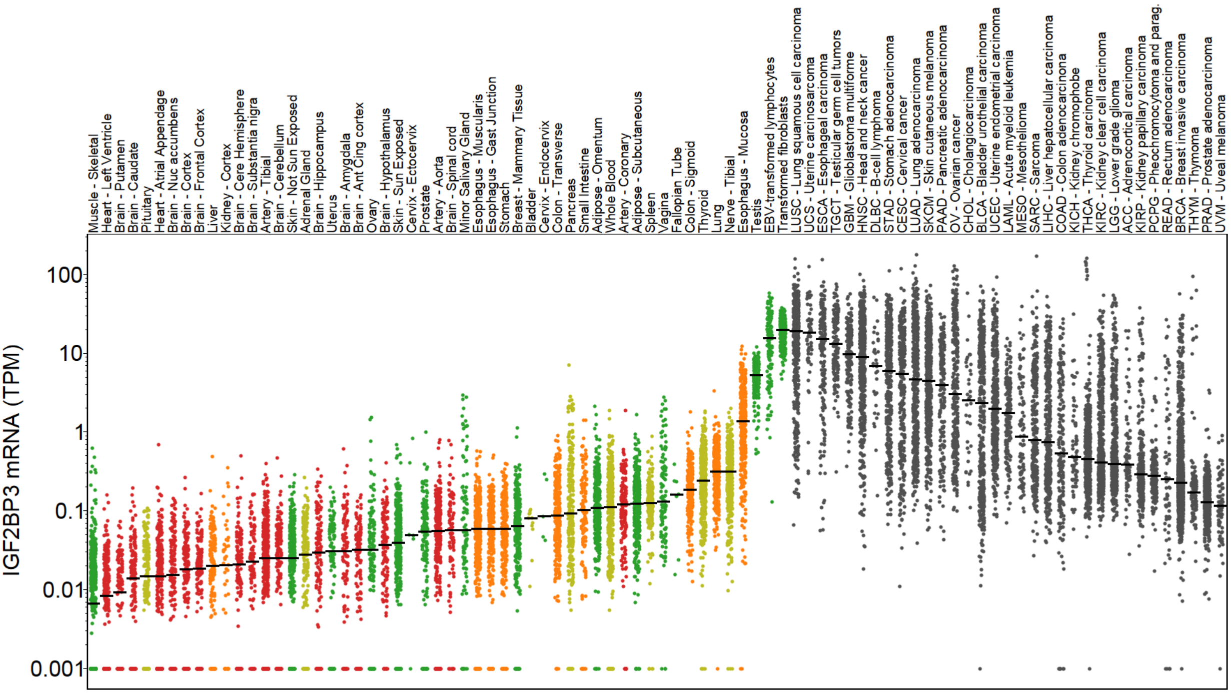

Supplementary Figure 10. Gene expression profile of IGF2BP3. Transcript expression of IGF2BP3 in normal tissues (colored dots, GTex Portal database) and human cancers (black dots, TCGA database), as determined by RNAseq analyses. Each dot represents one normal donor or patient tumor sample. Colors correspond to the four normal tissue categories defined in Figure 1B and Supplemental Table 2: Green, non-essential tissues; Yellow, caution tissues; Orange, hazard tissues; Red, danger tissues.

Supplementary Figure 11
GTex / TCGA expression profile – DEPDC1B

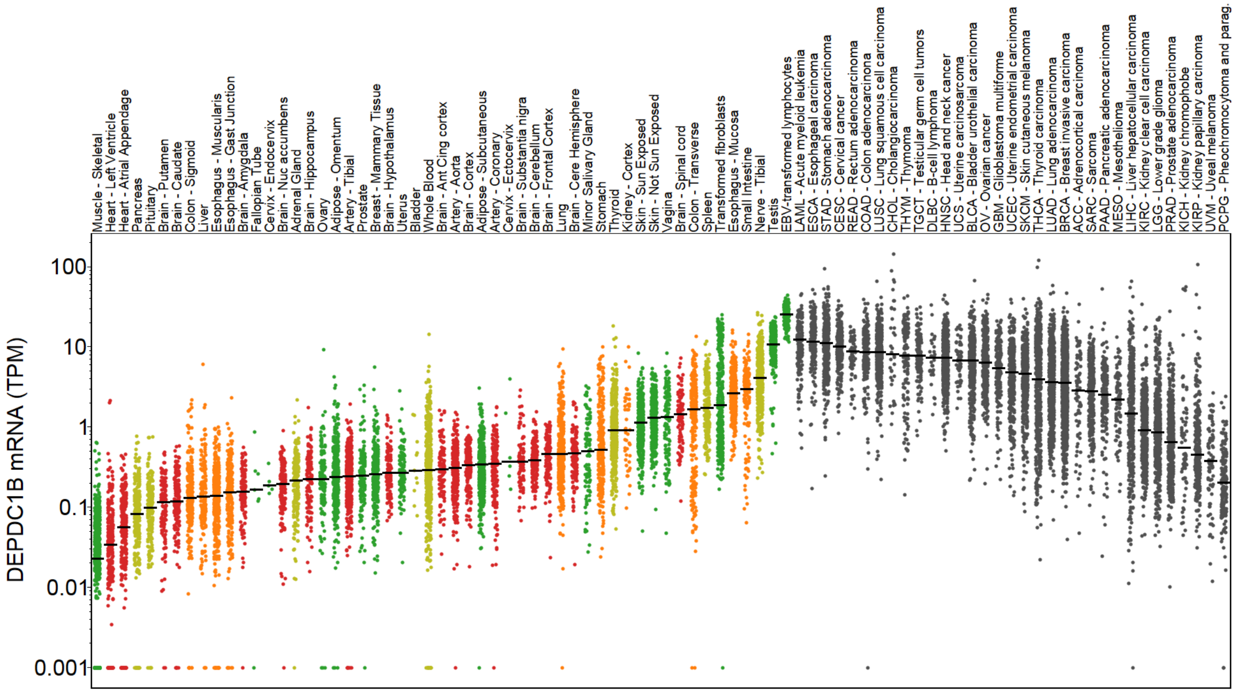

Supplementary Figure 11. Gene expression profile of DEPDC1B. Transcript expression of DEPDC1B in normal tissues (colored dots, GTex Portal database) and human cancers (black dots, TCGA database), as determined by RNAseq analyses. Each dot represents one normal donor or patient tumor sample. Colors correspond to the four normal tissue categories defined in Figure 1B and Supplemental Table 2: Green, non-essential tissues; Yellow, caution tissues; Orange, hazard tissues; Red, danger tissues.

Supplementary Figure 12
GTex / TCGA expression profile – ADAM12

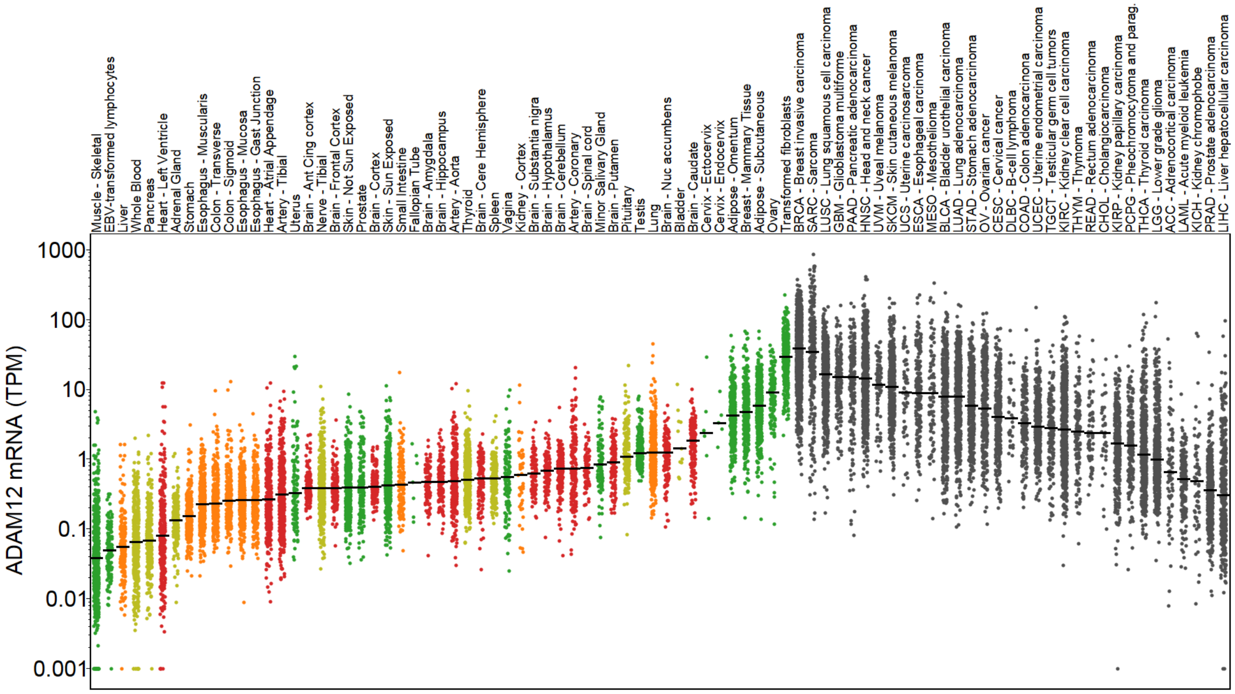

Supplementary Figure 12. Gene expression profile of ADAM12. Transcript expression of ADAM12 in normal tissues (colored dots, GTex Portal database) and human cancers (black dots, TCGA database), as determined by RNAseq analyses. Each dot represents one normal donor or patient tumor sample. Colors correspond to the four normal tissue categories defined in Figure 1B and Supplemental Table 2: Green, non-essential tissues; Yellow, caution tissues; Orange, hazard tissues; Red, danger tissues.

Supplementary Figure 13
GTex / TCGA expression profile – SLC38A9

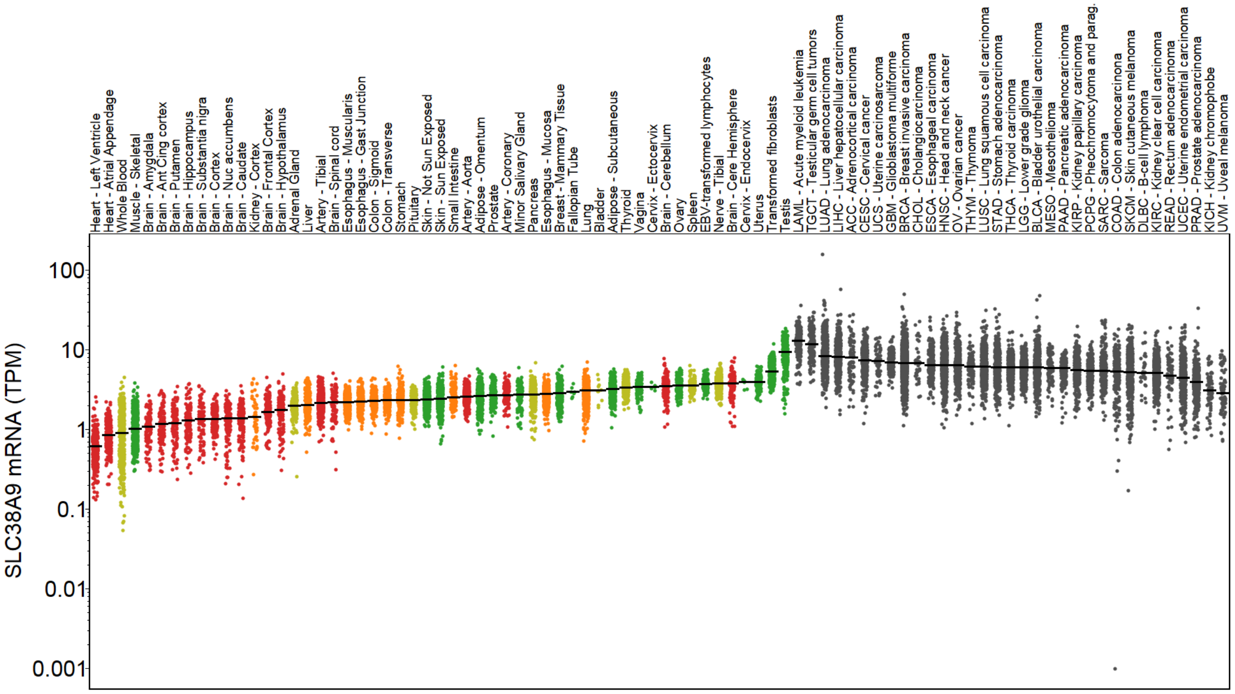

Supplementary Figure 13. Gene expression profile of SLC38A9. Transcript expression of SLC38A9 in normal tissues (colored dots, GTex Portal database) and human cancers (black dots, TCGA database), as determined by RNAseq analyses. Each dot represents one normal donor or patient tumor sample. Colors correspond to the four normal tissue categories defined in Figure 1B and Supplemental Table 2: Green, non-essential tissues; Yellow, caution tissues; Orange, hazard tissues; Red, danger tissues.

Supplementary Figure 14
GTex / TCGA expression profile – CAPN6

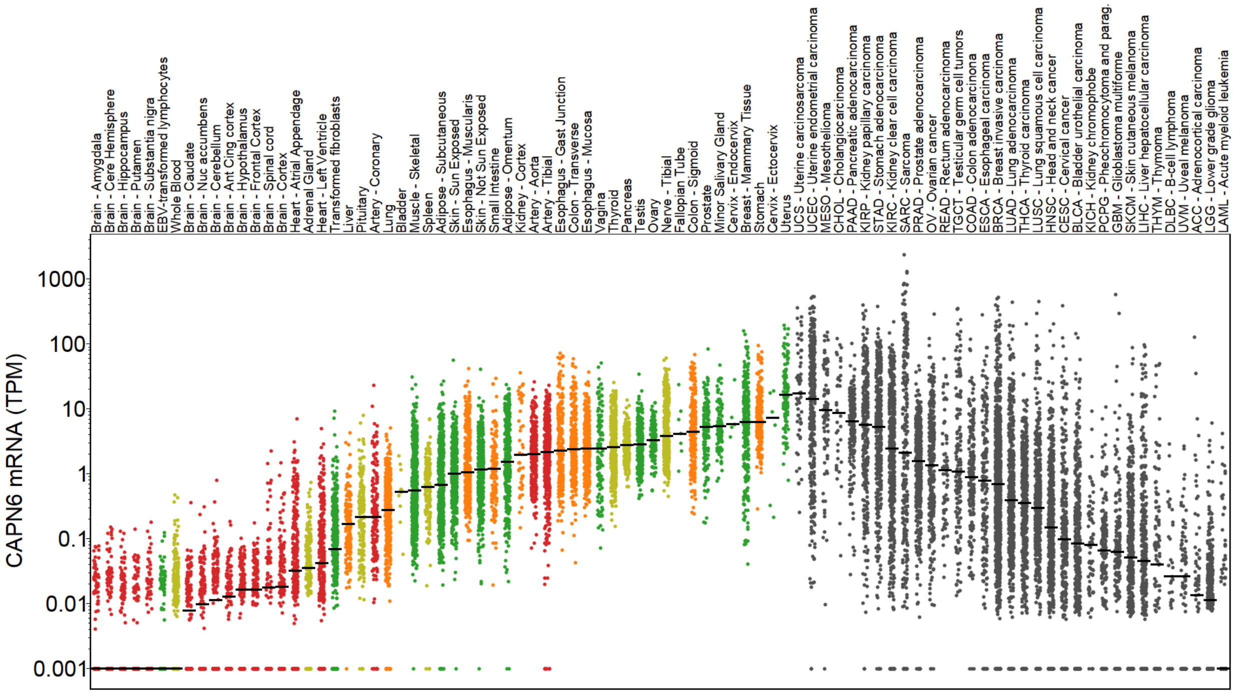

Supplementary Figure 14. Gene expression profiles of CAPN6. Transcript expression of CAPN6 in normal tissues (colored dots, GTex Portal database) and human cancers (black dots, TCGA database), as determined by RNAseq analyses. Each dot represents one normal donor or patient tumor sample. Colors correspond to the four normal tissue categories defined in Figure 1B and Supplemental Table 2: Green, non-essential tissues; Yellow, caution tissues; Orange, hazard tissues; Red, danger tissues.

Supplementary Figure 15
GTex / TCGA expression profile – MMP11

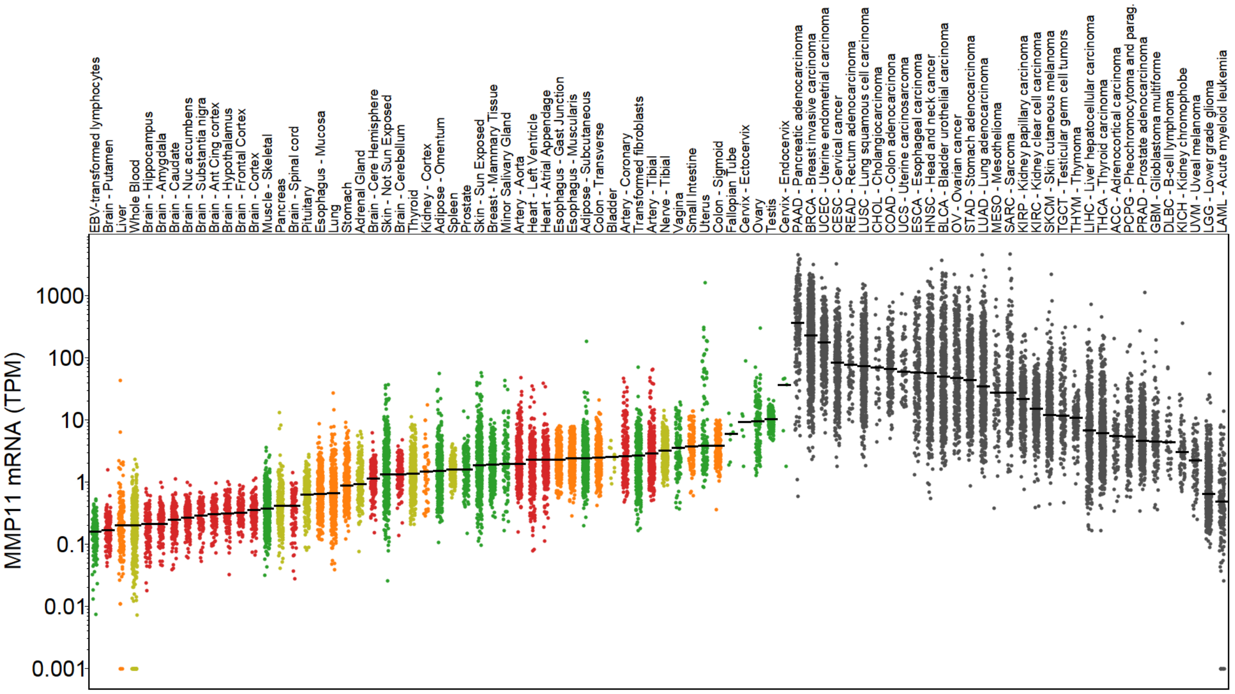

Supplementary Figure 15. Gene expression profiles of MMP11. Transcript expression of MMP11 in normal tissues (colored dots, GTex Portal database) and human cancers (black dots, TCGA database), as determined by RNAseq analyses. Each dot represents one normal donor or patient tumor sample. Colors correspond to the four normal tissue categories defined in Figure 1B and Supplemental Table 2: Green, non-essential tissues; Yellow, caution tissues; Orange, hazard tissues; Red, danger tissues.

Supplementary Figure 16.
VGLL1-specific CTLs were expanded from PBMC of multiple donors

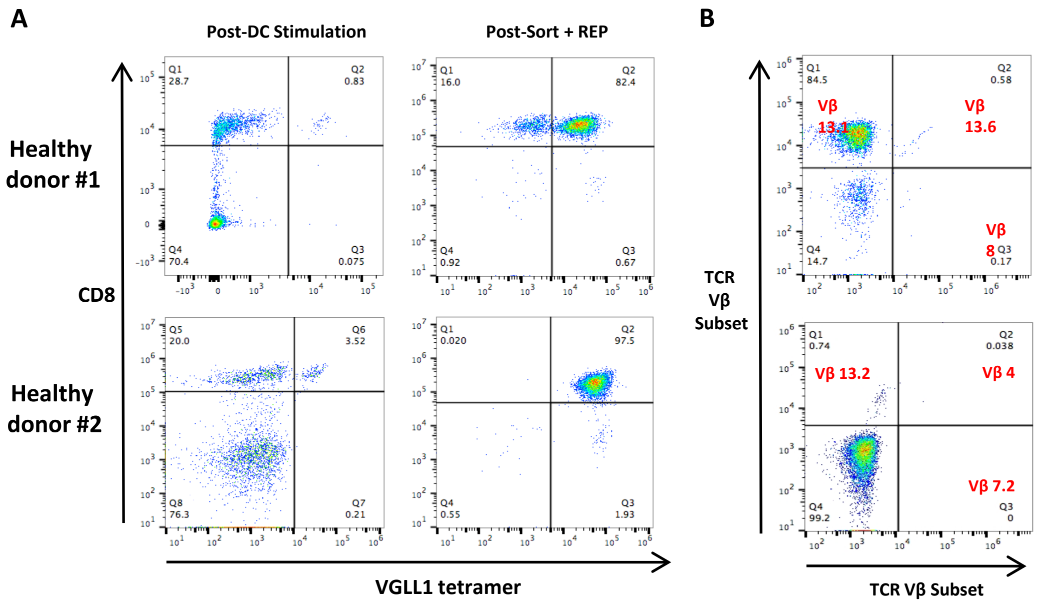

TCR Vb type	HD #1 (%)	HD #2 (%)
1	0.4	0
2	0	0
3	0	0.2
4	0	0
5.1	0	0.3
5.2	0	0.1
5.3	0.7	0
7.1	0.7	0
7.2	0	0
8	0	0
9	0.1	0
11	0	0
12	0	0
13.1	84.3	0
13.2	0	0.9
13.6	0.5	0
14	0	0.3
16	0	0
17	0	0
18	0	0.2
20	0	0
21.3	0	0
22	0	0
23	0	0.3

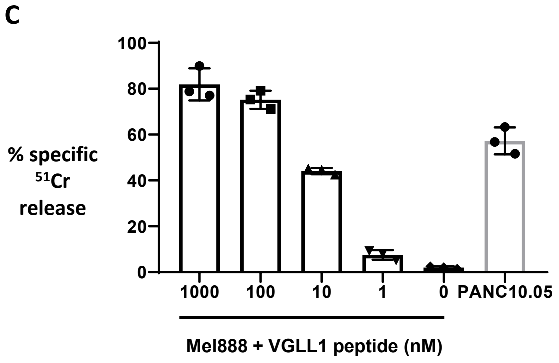

Supplementary Figure 16. Generation of HLA-A*0101-restricted VGLL1 antigen-specific CTLs from multiple normal donor PBMCs. (A) Induction of VGLL1-specific CD8 T cells from PBMCs of two healthy donors. HLA A*0101-expressing donor PBMCs were stimulated twice with LSELETPGKY peptide-pulsed dendritic cells over 2 weeks. VGLL1 tetramer-positive CD8+ T cells were sorted by ARIA sorter (top panels) and the sorted T cells were expanded using a standard rapid expansion protocol (REP). **(B)** TCR repertoire analysis of expanded VGLL1-specific CTLs was performed using Vβ antibodies corresponding to 24 different specificities. **(C)** VGLL1-specific T cells expanded from PBMCs of healthy donor #1 were tested for functionality in a standard ^{51}Cr release assay to assess specific lysis of Mel888 melanoma cells (VGLL1-negative HLA-A*0101-positive) pulsed with titrated amounts of LSELETPGKY peptide or pancreatic tumor cell line PANC10.05 at a 5:1 effector-to-target (E:T) ratio. Data shown is the mean and standard deviation of triplicate wells and is representative of two experiments that yielded similar results.

Supplementary Figure 17.
RNAseq analysis of lung tumor biopsies revealed loss of VGLL1 expression in PDAC Patient MP015

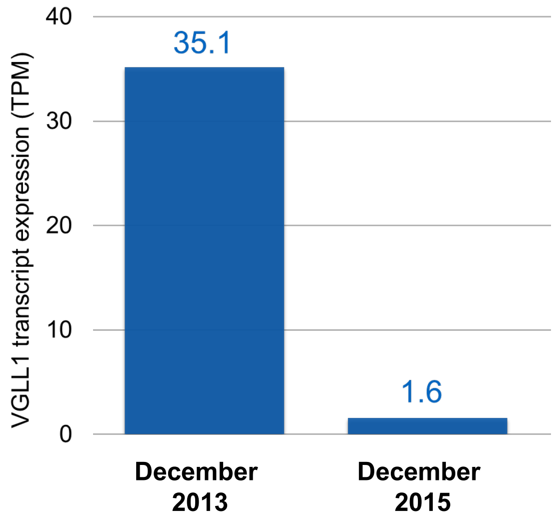

Supplementary Figure 17. PDAC patient MP015 showed loss of VGLL1 antigen expression prior to receiving VGLL1-specific CTLs. RNAseq analyses from lung tumor metastases of PDAC patient MP015 revealed loss of VGLL1 transcript expression between November 2013 and December 2015.

Supplementary Figure 18. HLA-A*0101 surface expression on tumor cells and primary cell lines

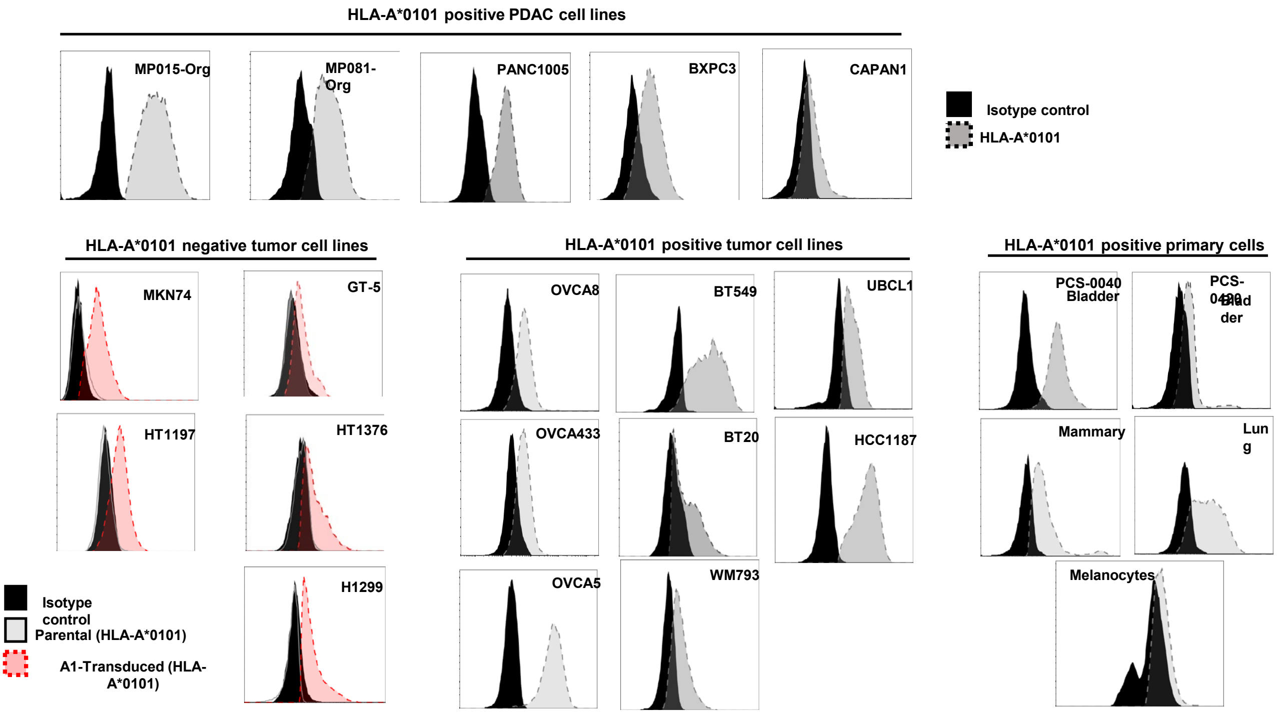

Supplementary Figure 18. HLA-A*0101 surface expression confirmed on target cell lines by flow cytometry. All tumor cell lines and normal primary cells used in this study were stained with fluorophore-labeled HLA-A*0101-specific mAb and analyzed by flow cytometry to confirm natural endogenous HLA-A*0101 surface expression (grey histograms) prior to use as targets for VGLL1-specific CTLs. Five tumor cell lines were transduced to express HLA-A*0101 using a lentiviral expression vector (red histograms).

Supplementary Figure 19.
HLA Class I blockade abrogates VGLL1-CTL recognition of PDAC cell line PANC10.05

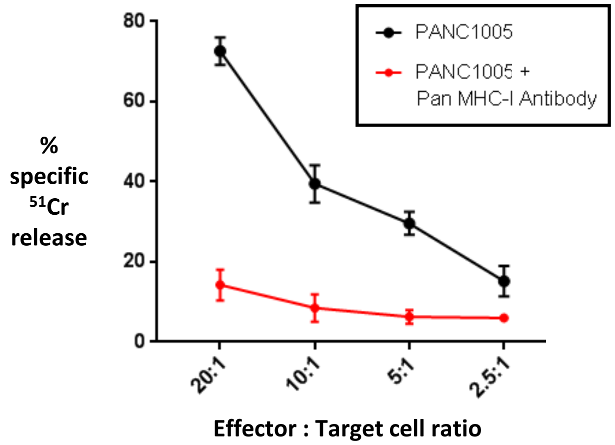

Supplementary Figure 19. Killing by VGLL1-specific CTLs is blocked with an HLA-class I-specific antibody. Expanded VGLL1-specific CD8+ T cells were co-cultured with HLA-A*0101-positive PDAC tumor cell line PANC10.05 in a standard ^{51}Cr release assay to measure cytotoxic activity at different effector-to-target (E:T) cell ratios. Addition of the HLA class I blocking antibody W6/32 largely abrogated target cell killing, demonstrating that antitumor activity is HLA class I-restricted. Data are presented as mean value +/- SD from 3 independent experiments.

Supplementary Figure 20
IFN-g treatment effect of VGLL1 T-cell killing of target cells

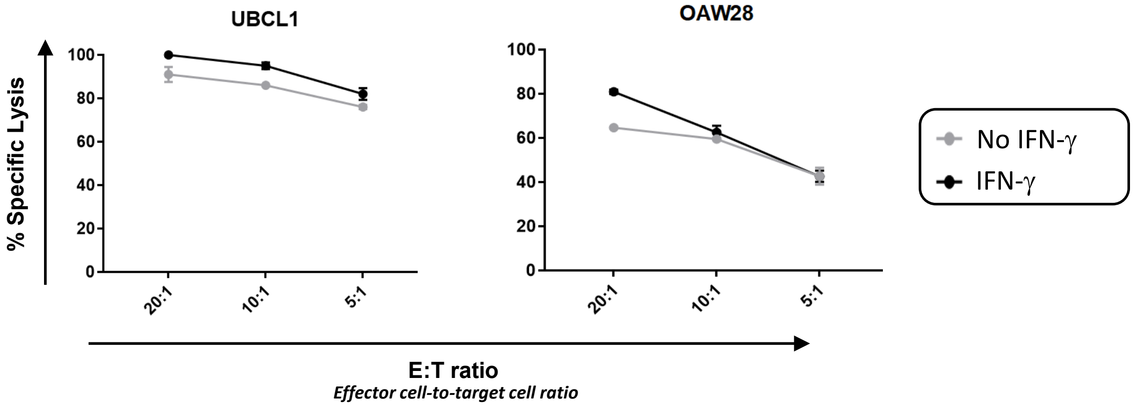

Supplementary Figure 20. IFN-γ treatment did not significantly affect tumor cell recognition by VGLL1-CTLs. Bladder tumor cell line UBCL1 and ovarian tumor cell line OVCAR5 were treated or not treated with 500U/mL human recombinant interferon-gamma for 48 hours prior to being exposed to VGLL1-CTLs at the indicated effector-to-target cell ratios. A standard ^{51}Cr release assay was used to measure tumor cell lysis. Data are presented as mean value +/- SD from 3 independent experiments.

Supplementary Figure 21
FACs gating strategy strategies

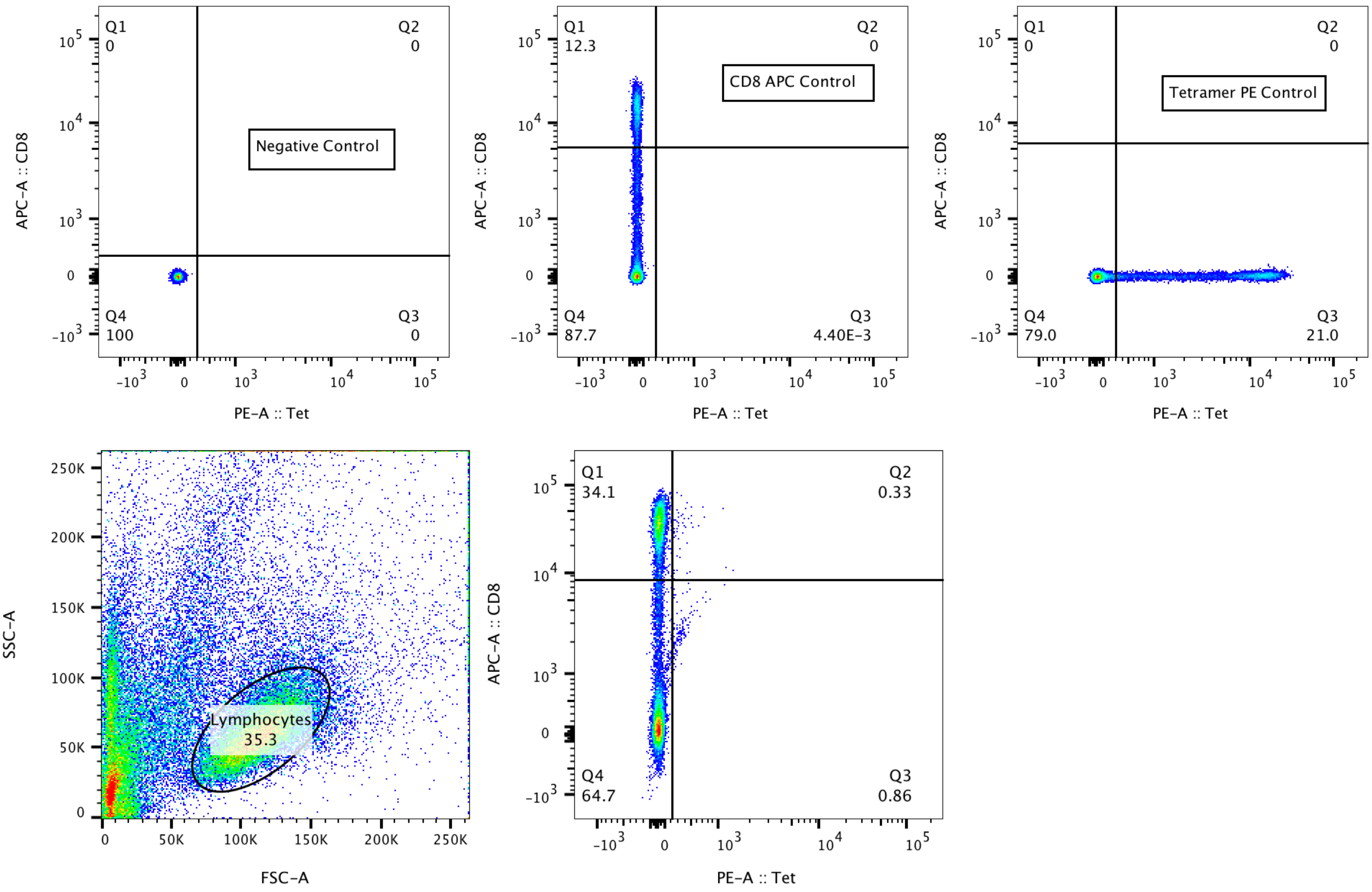

Supplementary Figure 21. Schematic depicting Flow Cytometry Gating Strategy for quantitating VGLL1 antigen-specific CD8+ T cells. Expanded T cells were lymphocyte gated as shown (bottom left) and stained with individual staining controls of anti-CD8-APC or HLA-A*0101/LSELETPGKY tetramer-PE (top). Tetramer-positive CD8+ T cells were detected by staining with both reagents (bottom right).

Supplementary Figure 22
Full Western Blot scans

A

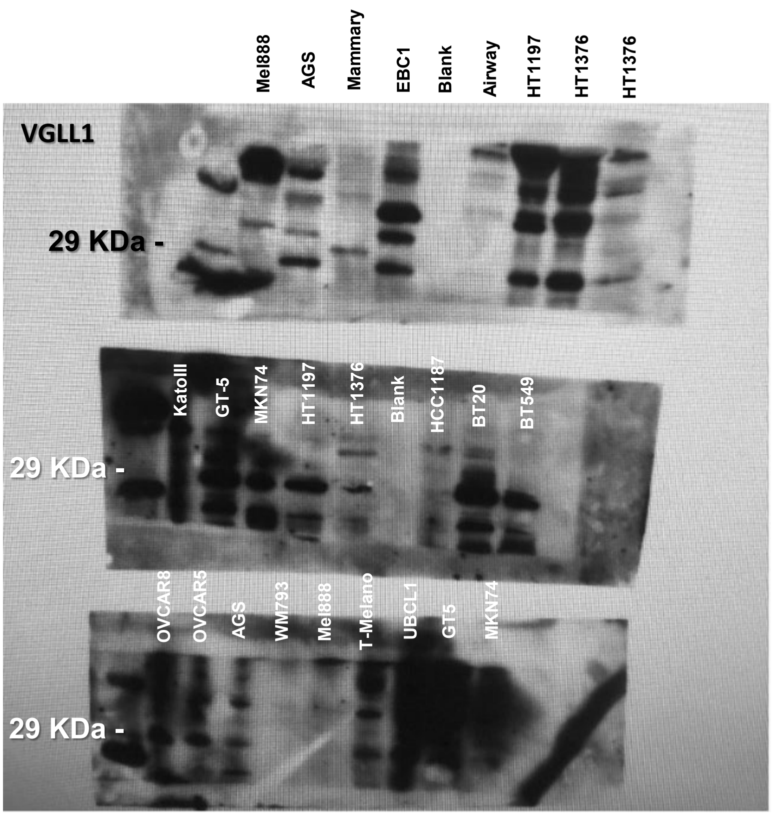

B

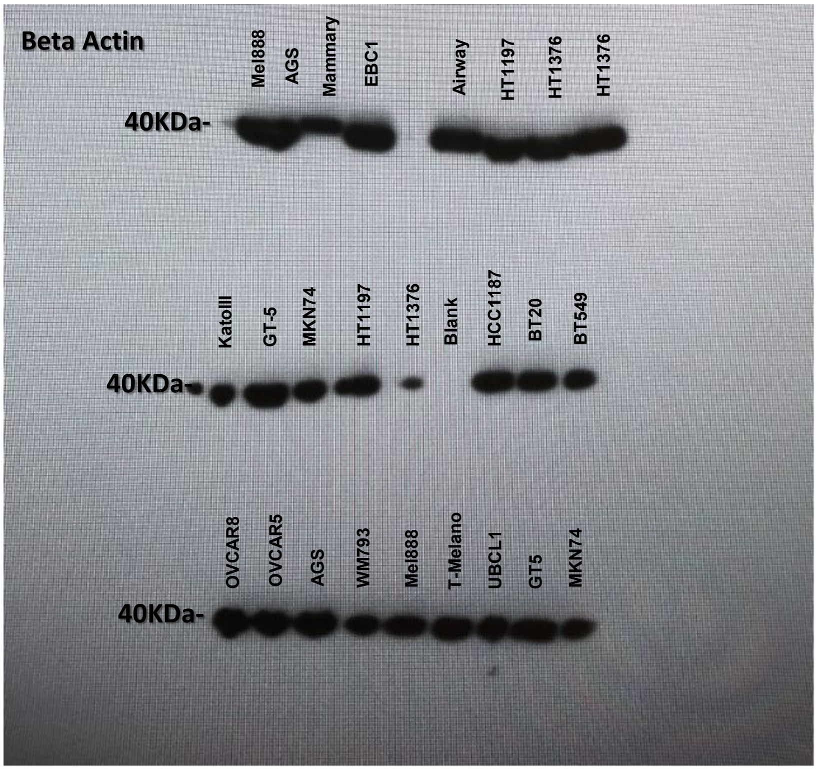

Supplementary Figure 22. Images of Full Western Blot Scans. (A) Representative Western blot analysis showing VGLL1 protein expression in primary and tumor cell lines derived from ovarian, lung, breast, bladder or gastric cancers.(B) Western blot analysis showing β-Actin protein expression in tumor cell lines derived from primary and tumor cell lines derived from ovarian, lung, breast, bladder or gastric cancers. These experiments were repeated three times with comparable results.

Supplementary Table 1

Tumor ID	PDAC Tumor type	Specimen type	Specimen quantity	HLA band intensity (W. blot)	Number of unique peptides (#1 rank)	% Potential binders to patient HLA*	# Putative TAA peptides	TAA Peptide sequence	TAA source gene	HLA class I restriction	Predicted HLA binding affinity (nM)**
MP06	Metastatic	Fresh tumor	230 mg	3	597	33.7	0				
MP010	Metastatic	Fresh tumor	60 mg	0	238	23.5	0				
MP012	Metastatic	Fresh tumor	120 mg	2	240	35.4	0				
MP012-X	Metastatic	Xenograft	230mg	2	565	34.5	0				
MP013	Metastatic	Fresh tumor	140 mg	3	1490	31.4	0				
MP015-Org	Metastatic	Organoid cell line	~90 million cells	4	1346	46.2	6	LSELETPGKY	VGLL1	A*0101	51
								LPHSEITTL		B*3502	385
								EVISSRGTSM	MUC16	A*2601	90
								THSTISQGF		B*3801	1836
								SPATAGPLLVLF		B*3502	1308
								KRFSHTHYI	ZNF717	B*3801 / C*1203	862 / 100
MP016	Metastatic	Fresh tumor	140 mg	3	750	37.7	0				
MP019	Metastatic	Fresh tumor	370mg	3	746	39.8	1	TPGGTRQSL	MUC16	B*0702	16
MP023	Metastatic	Fresh tumor	280mg	2	341	28.9	0				
MP025	Metastatic	Fresh tumor	90 mg	1	274	24.4	0				
MP025-X	Metastatic	Xenograft	320 mg	1	329	22.8	0				
MP031	Metastatic	Fresh tumor	180mg	3	1137	43.7	0				
MP037	Metastatic	Fresh tumor	15 mg	0	325	11.7	0				
MP041	Metastatic	Fresh tumor	150 mg	2	515	28.2	0				
MP041-X	Metastatic	Xenograft	200 mg	3	1663	39.8	0				
MP044	Primary	Fresh tumor	40 mg	2	586	27.1	0				
MP046	Primary	Fresh tumor	110 mg	2	955	35.3	0				
MP047	Primary	Fresh tumor	25 mg	1	383	24.8	0				
MP048	Primary	Fresh tumor	130 mg	2	440	26.1	1	TEITGLSAGV	MUC19	B*4501	735
MP050	Primary	Fresh tumor	170 mg	4	744	45.8	1	AVAIKAMAK	EIF5AL1	A*1101	32
MP054	Primary	Fresh tumor	60 mg	1	295	24.4	0				
MP055	Primary	Fresh tumor	320 mg	3	640	28.6	0				
MP056	Primary	Fresh tumor	60 mg	1	494	27.1	0				
MP058	Primary	Fresh tumor	20 mg	0	272	23.5	0				
MP063	Primary	Fresh tumor	30 mg	1	283	27.2	1	HEAERNIAL	RGPD1	B*1402 / C*0702	710 / 762
MP064	Metastatic	Fresh tumor	300 mg	1	794	29.1	0				
MP075	Metastatic	Fresh tumor	360 mg	2	259	32.8	0				
MP077	Metastatic	Fresh tumor	110 mg	2	503	36.8	1	ASEFQILKY	MIA2	A*0101	18
MP079	Metastatic	Fresh tumor	80 mg	2	326	35.6	0				
MP081	Metastatic	Fresh tumor	180 mg	2	392	18.1	0				
MP081-Org	Metastatic	Organoid cell line	~14 million cells	3	536	18.7	2	LSELETPGKY	VGLL1	A*0101	51
								ASEFQILKY	MIA2	A*0101	18
MP083	Metastatic	Fresh tumor	220 mg	1	277	20.6	0				
MP084	Metastatic	Fresh tumor	90 mg	2	374	36.6	0				
MP091	Metastatic	Fresh tumor	150 mg	2	335	32.2	0				
MP093	Metastatic	Fresh tumor	100 mg	1	326	42.6	1	QGKLSRMLSSY	SLC30A8	B*1501	602
MP098	Primary	Fresh tumor	110 mg	2	369	30.9	0				
MP099	Primary	Fresh tumor	190 mg	3	679	38.3	0				
MP104	Primary	Fresh tumor	80 mg	3	398	34.4	0				
MP108	Primary	Fresh tumor	180 mg	3	1657	43.8	0				
				HLA-low	22873 peptides	34.5 % binders	12 Unique TAAs				

*Predicted binding affinity <2000nM
** Determined by NetMHC3.4

Supplementary Table 2

DANGER tissues (1 TPM)	HAZARD tissues (3 TPM)	CAUTION tissues (10 TPM)	NON-ESSENTIAL tissues (30 TPM)	OTHER tissues (No TPM limit)
Artery - Aorta	Lung	Spleen	Breast - Mammary Tissue	Testis
Artery - Coronary	Nerve - Tibial	Pancreas	Cervix	* Placenta
Artery - Tibial	Colon - Sigmoid	Thyroid	Fallopian Tube	** EBV-transformed lymphocytes
Brain - Amygdala	Colon - Transverse	Whole Blood	Ovary	** Transformed fibroblasts
Brain - Cerebellar Hemisphere	Esophagus - GE Junction	Adrenal Gland	Prostate	
Brain - Cerebellum	Esophagus - Mucosa	Pituitary	Skin - Suprapubic	
Brain - Cortex	Esophagus - Muscularis		Uterus	* Non-GTex tissue RNAseq
Brain - Frontal Cortex	Kidney - Cortex		Vagina	** Transformed normal cells
Brain - Hippocampus	Liver		Adipose - Subcutaneous	
Brain - Hypothalamus	Small Intestine		Bladder	
Brain - Spinal cord (cervical c-1)	Stomach		Minor Salivary Gland	
Heart - Atrial Appendage			Muscle - Skeletal	
Heart - Left Ventricle				

Supplementary Table 3

GTex normal tissue CPA expression (mean TPM)	Normal Tissue	Number of samples	VGLL1	PLAC1	CGB3	CGB5	IGF2BP3	DEPDC1B	ADAM12	SLC38A9	CAPN6	MMP11
	Placenta	7	302.7	59.7	31.9	34.6	42.9	13.9	260.6	92.0	133.6	75.1
	Bladder	11	17.7	0.3	0.1	0.0	0.1	0.5	2.9	3.2	0.7	2.5
	Minor Salivary Gland	97	5.5	0.1	0.0	0.0	0.3	0.8	1.4	2.8	8.0	4.2
	Breast - Mammary Tissue	290	3.7	0.0	0.0	0.0	0.1	0.3	7.6	3.0	12.6	2.4
	Kidney - Cortex	45	2.0	0.0	0.0	0.0	0.0	1.6	1.1	1.7	6.8	2.4
	Prostate	152	1.7	0.0	0.1	0.0	0.1	0.4	0.7	2.8	7.8	2.1
	Esophagus - Mucosa	407	1.4	0.0	0.0	0.0	2.0	3.2	0.3	2.8	3.8	0.9
	Lung	427	1.3	0.1	0.1	0.0	0.4	0.7	2.0	3.1	0.5	1.1
	Vagina	115	1.1	0.1	0.1	0.0	0.4	1.8	0.9	3.5	5.8	4.7
	Fallopian Tube	7	0.4	0.2	0.0	0.0	0.2	0.3	0.7	3.0	7.3	6.1
	Cervix	6	0.0	0.1	0.0	0.0	0.1	0.2	4.3	3.8	10.2	28.0
	Uterus	111	0.1	0.1	0.1	0.0	0.0	0.4	1.6	4.1	31.9	40.0
	Ovary	133	0.0	0.2	0.0	0.0	0.1	0.4	11.3	3.7	4.0	15.6
	Testis	259	0.2	1.8	0.8	0.4	5.5	11.4	1.5	9.5	3.8	10.5
	Stomach	262	0.3	0.0	0.1	0.0	0.1	1.1	0.2	2.4	10.6	1.4
	Pancreas	248	0.3	0.0	0.3	0.0	0.3	0.1	0.1	2.9	3.1	0.6
	Skin - Suprapubic	387	0.2	0.1	0.1	0.0	0.0	1.5	0.6	2.5	2.0	2.7
	Spleen	162	0.0	0.1	0.1	0.0	0.2	2.4	0.6	3.7	0.9	1.7
	Whole Blood	407	0.0	0.0	0.0	0.0	0.2	0.6	0.1	1.0	0.0	0.3
	Muscle - Skeletal	564	0.0	0.1	0.0	0.0	0.0	0.0	0.1	1.2	1.6	0.5
	Adipose - Subcutaneous	442	0.0	0.0	0.0	0.0	0.2	0.4	7.6	3.3	1.8	3.6
	Thyroid	446	0.0	0.0	0.0	0.0	0.3	1.3	0.7	3.5	3.9	1.8
	Liver	175	0.0	0.0	0.0	0.0	0.0	0.2	0.1	2.2	0.3	0.6
	Esophagus - GE Junction	244	0.0	0.0	0.0	0.0	0.1	0.2	0.4	2.4	7.4	2.7
	Small Intestine	137	0.0	0.0	0.1	0.0	0.2	3.3	0.8	2.8	2.4	4.6
	Colon - Transverse	274	0.0	0.0	0.0	0.0	0.1	2.1	0.4	2.4	5.4	3.3
	Brain - Frontal Cortex	129	0.0	0.0	0.0	0.0	0.0	0.5	0.5	1.8	0.0	0.4
	Brain - Cerebellum	173	0.0	0.0	0.0	0.0	0.0	0.4	0.8	3.5	0.0	1.5
	Artery - Aorta	299	0.0	0.1	0.0	0.0	0.1	0.4	0.8	2.6	3.0	3.4
	Artery - Coronary	173	0.0	0.0	0.0	0.0	0.2	0.4	1.6	2.8	0.8	4.9
	Heart - Atrial Appendage	297	0.0	0.0	0.0	0.0	0.0	0.1	0.6	0.9	0.2	2.9
	Heart - Left Ventricle	303	0.0	0.0	0.0	0.0	0.0	0.1	0.4	0.6	0.2	2.9
	Transformed fibroblasts	343	0.0	0.9	0.1	0.0	20.3	4.7	36.4	5.7	0.3	3.6
	EBV-transformed lymphocytes	130	0.0	0.1	0.0	0.0	17.7	25.8	0.1	3.9	0.0	0.2

Supplementary Table 4

Mean CPA transcript expression of Ag-positive TCGA tumors (>5 TPM)	Tumor ID	Cancer Type	Total number of samples	VGLL1	PLAC1	CGB3	CGB5	IGF2BP3	DEPDC1B	ADAM12	SLC38A9	CAPN6	MMP11
	BLCA	Bladder urothelial carcinoma	408	124.5	12.5	120.9	83.0	16.7	10.4	24.7	8.6	26.3	209.1
	BRCA Basal-like	Breast carcinoma (basal-like)	134	118.5	7.1	0.0	0.0	15.3	10.3	36.5	7.8	68.7	219.6
	OV	Ovarian serous cystadenocarcinoma	307	37.7	22.3	6.1	0.0	29.3	11.2	24.6	8.9	22.6	188.3
	PAAD	Pancreatic adenocarcinoma	179	21.7	0.0	111.0	88.1	12.7	7.1	29.0	6.7	14.2	662.3
	CHOL	Cholangiocarcinoma	36	24.3	0.0	12.1	8.6	14.5	18.8	10.4	9.5	40.9	126.9
	UCS	Uterine carcinosarcoma	57	12.9	19.3	10.5	7.5	26.1	9.2	22.2	8.7	65.9	118.6
	CESC	Cervical and endocervical cancers	306	22.9	10.8	32.7	23.7	18.4	11.9	16.6	8.6	43.4	171.4
	LUSC	Lung squamous cell carcinoma	501	23.4	13.8	23.5	28.0	27.7	10.6	26.1	8.1	37.1	153.5
	UCEC	Uterine corpus endometrial carcinoma	370	17.9	15.4	27.2	19.0	18.4	11.1	11.9	7.8	67.6	236.8
	LUAD	Lung adenocarcinoma	517	26.6	10.0	39.6	36.2	23.3	10.6	20.7	10.4	33.1	129.4
	THCA	Thyroid carcinoma	509	9.9	0.0	5.0	6.4	80.5	12.4	19.8	7.0	17.3	25.7
	HNSC	Head and neck squamous cell carcinoma	522	15.4	11.3	31.4	22.5	19.7	10.6	34.8	8.4	32.7	155.0
	STAD	Stomach adenocarcinoma	378	31.7	8.4	21.7	43.6	19.9	14.4	16.9	8.4	33.2	113.9
	TGCT	Testicular germ cell tumors	156	14.7	9.4	116.1	97.8	20.7	10.7	11.6	12.8	64.1	37.1
	ESCA	Esophageal carcinoma	185	24.6	10.0	11.9	11.4	26.3	15.0	29.1	8.5	27.1	163.4
	BRCA Non-basal	Breast carcinoma (non-basal)	966	45.6	11.5	5.4	8.1	11.4	8.8	59.9	8.7	28.6	387.4
	PRAD	Prostate adenocarcinoma	498	8.8	0.0	0.0	0.0	0.0	9.6	16.3	6.7	9.5	19.1
	THYM	Thymoma	119	8.9	0.0	8.3	10.7	34.3	11.9	14.7	7.3	31.9	16.9
	KIRP	Kidney renal papillary cell carcinoma	291	47.5	10.5	0.0	0.0	15.5	9.3	9.6	7.3	41.3	32.4
	COAD	Colon adenocarcinoma	191	12.4	6.4	31.7	30.7	10.6	11.9	11.3	8.1	18.3	113.5
	READ	Rectum adenocarcinoma	72	5.8	10.2	16.7	15.2	11.4	8.8	14.4	7.6	0.0	138.6
	LIHC	Liver hepatocellular carcinoma	373	16.4	6.6	8.8	9.2	14.1	15.1	15.6	10.1	29.1	29.9
	KIRC	Kidney renal clear cell carcinoma	533	12.7	0.0	21.6	14.6	13.3	6.3	17.4	6.9	23.9	21.0
	ACC	Adrenocortical carcinoma	79	0.0	54.0	7.8	0.0	12.3	10.9	16.2	11.2	57.8	15.0
	DLBC	Diffuse large B-cell Lymphoma	33	0.0	0.0	0.0	0.0	15.9	12.9	17.2	8.3	0.0	18.5
	GBM	Glioblastoma multiforme	166	0.0	28.0	0.0	0.0	16.3	9.4	23.2	7.7	23.3	16.1
	KICH	Kidney chromophobe	66	0.0	0.0	0.0	0.0	9.3	0.0	35.0	6.6	9.4	34.0
	LAML	Acute myeloid leukemia	173	0.0	0.0	0.0	0.0	8.5	12.4	8.7	13.7	0.0	7.7
	LGG	Brain lower grade glioma	530	0.0	0.0	0.0	0.0	16.8	10.7	17.0	6.9	6.1	11.8
	MESO	Mesothelioma	87	0.0	18.3	8.2	11.2	12.4	7.8	42.7	7.6	35.5	73.1
	PCPG	Pheochromocytoma and paraganglioma	184	0.0	0.0	17.2	7.8	0.0	0.0	10.8	7.0	9.0	18.0
	SARC	Sarcoma	263	0.0	22.2	22.9	39.3	16.2	7.9	89.6	8.2	170.2	129.3
	SKCM	Skin Cutaneous Melanoma	472	0.0	10.7	12.5	7.4	16.0	9.0	24.1	7.9	22.4	38.1
	UVM	Uveal melanoma	80	0.0	0.0	0.0	0.0	0.0	0.0	18.1	7.3	0.0	8.1
	BRCA	Breast invasive carcinoma	1100	80.9	11.4	5.4	8.1	14.2	9.2	57.2	8.6	42.1	367.1

Supplementary Table 5

Percentage of TCGA tumors with CPA transcript > 5 TPM	Tumor ID	Cancer Type	Total number of samples	VGLL1	PLAC1	CGB3	CGB5	IGF2BP3	DEPDC1B	ADAM12	SLC38A9	CAPN6	MMP11
	BLCA	Bladder urothelial carcinoma	408	82.4	19.4	15.0	11.3	40.2	41.2	59.1	67.4	5.6	92.9
	BRCA Basal-like	Breast carcinoma (basal-like)	134	82.2	3.0	0.0	0.0	26.9	74.6	89.6	59.7	49.3	99.3
	OV	Ovarian serous cystadenocarcinoma	307	73.9	10.4	1.3	0.0	43.3	31.9	51.5	72.3	25.4	96.4
	PAAD	Pancreatic adenocarcinoma	179	46.4	0.0	41.9	40.2	45.8	13.4	77.7	72.1	58.1	98.9
	CHOL	Cholangiocarcinoma	36	30.6	0.0	11.1	11.1	33.3	19.4	27.8	75.0	58.3	97.2
	UCS	Uterine carcinosarcoma	57	29.8	35.1	3.5	3.5	89.5	57.9	71.9	75.4	78.9	100.0
	CESC	Cervical and endocervical cancers	306	28.4	17.0	6.9	4.2	52.0	41.8	43.1	83.3	7.8	98.4
	LUSC	Lung squamous cell carcinoma	501	28.3	18.8	3.6	2.2	79.8	68.1	84.8	71.3	7.2	98.2
	UCEC	Uterine corpus endometrial carcinoma	370	27.0	2.2	20.5	20.5	30.3	14.1	30.0	39.2	67.0	100.0
	LUAD	Lung adenocarcinoma	517	21.5	7.4	5.0	4.1	49.7	29.6	63.1	90.1	14.7	93.0
	THCA	Thyroid carcinoma	509	18.7	0.0	0.2	0.2	2.6	11.0	15.5	74.7	5.3	60.1
	HNSC	Head and neck squamous cell carcinoma	522	18.0	22.4	40.5	9.4	66.1	60.2	80.5	74.7	4.4	95.0
	STAD	Stomach adenocarcinoma	378	17.7	4.2	3.7	4.8	54.0	47.6	54.8	67.5	51.6	96.0
	TGCT	Testicular germ cell tumors	156	14.7	14.7	39.7	33.3	85.9	20.5	26.3	98.1	30.1	85.9
	ESCA	Esophageal carcinoma	185	13.5	10.3	4.9	4.9	73.5	64.9	68.1	74.1	23.8	95.1
	BRCA Non-basal	Breast carcinoma (non-basal)	966	12.1	13.0	0.2	0.2	1.8	29.9	95.4	81.5	13.3	99.6
	PRAD	Prostate adenocarcinoma	498	6.4	0.0	0.0	0.0	0.0	0.4	0.8	26.3	17.3	46.6
	THYM	Thymoma	119	4.2	0.0	5.0	2.5	7.6	12.6	28.6	73.9	5.9	88.2
	KIRP	Kidney renal papillary cell carcinoma	291	2.7	0.3	0.0	0.0	7.2	1.0	16.8	62.9	52.6	92.4
	COAD	Colon adenocarcinoma	191	2.6	2.1	4.7	4.7	19.4	35.1	39.3	55.5	20.4	100.0
	READ	Rectum adenocarcinoma	72	1.4	5.6	5.6	6.9	18.1	20.8	25.0	48.6	0.0	100.0
	LIHC	Liver hepatocellular carcinoma	373	1.3	0.3	0.8	0.8	24.7	2.9	6.4	86.1	7.2	60.6
	KIRC	Kidney renal clear cell carcinoma	533	0.8	0.0	0.4	0.2	13.3	1.7	36.0	56.5	37.3	93.4
	ACC	Adrenocortical carcinoma	79	0.0	11.4	1.3	0.0	8.9	6.3	11.4	73.4	3.8	53.2
	DLBC	Diffuse large B-cell Lymphoma	33	0.0	0.0	0.0	0.0	60.6	36.4	42.4	54.5	0.0	45.5
	GBM	Glioblastoma multiforme	166	0.0	1.2	0.0	0.0	74.1	42.8	83.7	91.0	3.0	46.4
	KICH	Kidney chromophobe	66	0.0	0.0	0.0	0.0	12.1	0.0	6.1	18.2	1.5	24.2
	LAML	Acute myeloid leukemia	173	0.0	0.0	0.0	0.0	13.9	0.6	0.6	97.1	0.0	4.6
	LGG	Brain lower grade glioma	530	0.0	0.0	0.0	0.0	10.6	3.8	14.2	79.4	0.2	4.5
	MESO	Mesothelioma	87	0.0	2.3	2.3	2.3	19.5	13.8	62.1	69.0	67.8	89.7
	PCPG	Pheochromocytoma and paraganglioma	184	0.0	0.0	0.5	0.5	0.0	0.0	18.5	59.8	1.1	56.0
	SARC	Sarcoma	263	0.0	17.9	4.2	3.4	23.2	25.5	84.8	59.7	44.9	94.7
	SKCM	Skin Cutaneous Melanoma	472	0.0	2.5	0.6	0.4	48.5	36.9	74.4	55.9	2.8	83.7
	UVM	Uveal melanoma	80	0.0	0.0	0.0	0.0	0.0	0.0	77.5	25.0	0.0	10.0
	BRCA	Breast invasive carcinoma	1100	20.6	11.8	0.2	0.2	4.7	33.9	94.7	78.8	17.7	99.5

Supplementary Table 6

CPA transcript expression of all TCGA tumors (mean TPM)	Tumor ID	Cancer Type	Total number of samples	VGLL1	PLAC1	CGB3	CGB5	IGF2BP3	DEPDC1B	ADAM12	SLC38A9	CAPN6	MMP11
	BLCA	Bladder urothelial carcinoma	408	102.8	3.3	18.2	15.8	7.3	8.0	15.4	7.0	1.8	194.4
	BRCA Basal-like	Breast carcinoma (basal-like)	134	97.6	0.9	0.1	0.1	4.9	8.6	33.1	6.0	34.4	217.9
	OV	Ovarian serous cystadenocarcinoma	307	28.4	3.0	0.3	0.1	13.4	8.4	13.7	7.5	6.7	181.7
	PAAD	Pancreatic adenocarcinoma	179	10.6	0.4	47.4	36.2	6.7	3.5	23.0	5.9	9.2	651.3
	CHOL	Cholangiocarcinoma	36	7.8	0.1	1.5	1.0	5.8	21.5	4.2	8.1	24.6	123.5
	LUSC	Lung squamous cell carcinoma	501	7.6	3.6	2.4	1.0	22.5	9.7	22.5	6.9	4.1	150.8
	CESC	Cervical and endocervical cancers	306	7.3	2.8	3.0	1.6	10.2	11.2	8.4	7.8	3.8	168.6
	LUAD	Lung adenocarcinoma	517	6.6	1.5	6.6	6.0	12.3	5.5	13.9	9.8	5.4	120.6
	STAD	Stomach adenocarcinoma	378	6.3	0.9	4.2	3.9	11.4	13.3	10.2	6.9	17.8	109.5
	BRCA Non-basal	Breast carcinoma (non-basal)	966	6.1	2.8	0.2	0.2	0.5	4.3	57.3	7.8	4.6	385.8
	UCEC	Uterine corpus endometrial carcinoma	370	5.7	1.6	6.5	6.9	6.6	6.1	5.2	5.1	45.8	236.8
	UCS	Uterine carcinosarcoma	57	4.8	7.8	3.5	1.6	23.5	7.8	16.6	7.5	52.5	118.6
	ESCA	Esophageal carcinoma	185	4.4	2.0	0.9	0.9	19.8	14.2	20.6	7.3	7.1	155.6
	HNSC	Head and neck squamous cell carcinoma	522	3.8	3.7	5.4	2.8	13.7	8.6	28.5	7.2	1.9	147.9
	THCA	Thyroid carcinoma	509	2.7	0.1	0.1	0.1	2.8	7.0	4.2	6.3	1.6	16.5
	TGCT	Testicular germ cell tumors	156	2.6	2.5	57.4	33.3	18.2	9.1	4.6	12.7	20.0	32.3
	PRAD	Prostate adenocarcinoma	498	1.6	0.1	0.1	0.1	0.2	1.2	0.6	4.3	2.9	10.5
	KIRP	Kidney renal papillary cell carcinoma	291	1.5	0.1	0.0	0.0	1.6	1.7	3.0	6.0	22.5	30.2
	THYM	Thymoma	119	1.0	0.1	1.0	1.0	2.8	10.6	5.5	6.4	2.1	15.2
	COAD	Colon adenocarcinoma	191	0.5	0.8	1.9	1.9	2.8	10.3	5.7	5.8	4.6	113.5
	READ	Rectum adenocarcinoma	72	0.4	1.4	6.7	6.3	2.5	8.9	5.2	5.5	4.8	138.6
	LIHC	Liver hepatocellular carcinoma	373	0.3	0.1	0.1	0.1	4.2	3.7	1.6	9.2	2.4	18.9
	KIRC	Kidney renal clear cell carcinoma	533	0.2	0.1	0.1	0.1	2.4	1.3	7.4	5.5	9.7	19.6
	SKCM	Skin Cutaneous Melanoma	472	0.1	0.7	0.2	0.1	8.7	5.5	18.7	5.9	0.8	32.4
	SARC	Sarcoma	263	0.0	4.4	18.4	6.7	4.6	3.7	76.3	6.5	76.8	122.6
	ACC	Adrenocortical carcinoma	79	0.0	6.6	0.2	0.0	1.7	4.5	2.6	9.3	2.4	9.1
	DLBC	Diffuse large B-cell Lymphoma	33	0.0	0.2	0.1	0.0	10.5	8.5	8.6	6.2	0.2	9.9
	GBM	Glioblastoma multiforme	166	0.0	0.7	0.0	0.0	12.8	6.6	19.9	7.4	6.2	9.1
	KICH	Kidney chromophobe	66	0.0	0.1	0.1	0.0	1.8	4.3	2.8	3.5	0.4	10.1
	LAML	Acute myeloid leukemia	173	0.0	0.1	0.0	0.0	2.7	13.4	0.7	13.4	0.1	1.2
	LGG	Brain lower grade glioma	530	0.0	0.2	0.0	0.0	2.4	1.9	3.3	6.3	0.1	1.5
	MESO	Mesothelioma	87	0.0	0.7	0.4	0.4	3.3	2.9	27.4	6.4	24.6	65.9
	PCPG	Pheochromocytoma and paraganglioma	184	0.0	0.0	0.1	0.1	0.4	0.3	3.3	5.7	0.3	11.3
	UVM	Uveal melanoma	80	0.0	0.0	0.0	0.0	0.2	0.6	14.5	3.7	0.2	2.7
	BRCA	Breast invasive carcinoma	1100	17.2	2.5	0.2	0.2	1.1	4.8	54.4	7.6	8.2	365.4
