# Supplementary material for: Vestigial-like 1 is a shared targetable cancer-placenta antigen expressed by pancreatic and basal-like breast cancers
Source: Nat Commun. 2020 Oct 21;11:5332. doi: 10.1038/s41467-020-19141-w (PMC7577998; doi:10.1038/s41467-020-19141-w)
Supplement: Supplementary file 2 — Description of Additional Supplementary Files [file 41467_2020_19141_MOESM2_ESM.docx]

**Description of Additional Supplementary Files**

File Name: Supplementary Data 1

Description: A table containing all the peptides eluted from MP015 tumor, MS parameters and BLAST search results, predicted HLA-peptide binding affinities (nM), Tumor RNAseq, GTex Portal Normal Tissue RNAseq data, GTex Portal maximums and TCGA tumor RNAseq data.

File Name: Supplementary Data 2

Description: A table containing all the peptides eluted from MP081 tumor, MS parameters and BLAST search results, predicted HLA-peptide binding affinities (nM), Tumor RNAseq, GTex Portal Normal Tissue RNAseq data, GTex Portal maximums and TCGA tumor RNAseq data.
