# Supplementary material for: Vestigial-like 1 is a shared targetable cancer-placenta antigen expressed by pancreatic and basal-like breast cancers
Source: Nat Commun. 2020 Oct 21;11:5332. doi: 10.1038/s41467-020-19141-w (PMC7577998; doi:10.1038/s41467-020-19141-w)
Supplement: Supplementary file 5 — Reporting Summary [file 41467_2020_19141_MOESM5_ESM.pdf]

nature research

Corresponding author(s): Cassian Yee and Gregory Lizee

Last updated by author(s): 08/28/2020

Reporting Summary

Nature Research wishes to improve the reproducibility of the work that we publish. This form provides structure for consistency and transparency in reporting. For further information on Nature Research policies, see Authors & Referees and the Editorial Policy Checklist .

Statistics

For all statistical analyses, confirm that the following items are present in the figure legend, table legend, main text, or Methods section.

n/a	Confirmed	
☐	☒	The exact sample size (*n*) for each experimental group/condition, given as a discrete number and unit of measurement
☐	☒	A statement on whether measurements were taken from distinct samples or whether the same sample was measured repeatedly
☐	☒	The statistical test(s) used AND whether they are one- or two-sided<br>*Only common tests should be described solely by name; describe more complex techniques in the Methods section.*
☐	☒	A description of all covariates tested
☐	☒	A description of any assumptions or corrections, such as tests of normality and adjustment for multiple comparisons
☐	☒	A full description of the statistical parameters including central tendency (e.g. means) or other basic estimates (e.g. regression coefficient) AND variation (e.g. standard deviation) or associated estimates of uncertainty (e.g. confidence intervals)
☐	☒	For null hypothesis testing, the test statistic (e.g. *F*, *t*, *r*) with confidence intervals, effect sizes, degrees of freedom and *P* value noted<br>*Give P values as exact values whenever suitable.*
☒	☐	For Bayesian analysis, information on the choice of priors and Markov chain Monte Carlo settings
☒	☐	For hierarchical and complex designs, identification of the appropriate level for tests and full reporting of outcomes
☐	☒	Estimates of effect sizes (e.g. Cohen's *d*, Pearson's *r*), indicating how they were calculated

Our web collection on statistics for biologists contains articles on many of the points above.

Software and code

Policy information about availability of computer code

Data collection	RNAseq: Whole transcriptome sequencing (RNAseq) analysis was<br>performed on RNA derived from all PDAC tumor specimens, xenografts, and organoid cell lines using the<br>Illumina TruSeq Stranded Total RNA kit with Ribo-Zero Gold with approximately 200 million paired-end reads<br>for each tumor RNA sample (Avera Institute for Human Genetics).<br>Mass spectrometry: The acquired HLA class I-bound peptide mass spectra were analyzed by Proteome Discoverer 2.2. Mascot 2.6.2 was used for protein identification. The parameters used in search were: 10ppm for precursor mass tolerance, 0.8 Da for fragment mass tolerance, no specific enzyme digestion, and oxidation at methionine.<br>Flow Cytometry: Data was acquired using FACSDIVA software (version 8.0.1) and later analyzed using FlowJoTM software (version7.6.5).
Data analysis	Individual peptide matches underwent quality assessment by reference to multiple orthogonal<br>parameters, including Mascot Ion score, MS1 measured differential to the calculated peptide mass (delta mass),<br>and predicted binding to the patient’s HLA allotypes as determined by high-resolution genetic sequencing and<br>the NetMHC and NetMHCpan algorithms. High-confidence peptide matches were analyzed by BLAST (version BLAST+2.2.31)<br>searches to identify all potential source genes, which were then cross-referenced to RNAseq data derived from<br>individual tumor samples to provide further validation of peptide identity. Statistical analysis was performed using GraphPad Prism<br>(version 7.03) software.

For manuscripts utilizing custom algorithms or software that are central to the research but not yet described in published literature, software must be made available to editors/reviewers. We strongly encourage code deposition in a community repository (e.g. GitHub). See the Nature Research guidelines for submitting code & software for further information.

Data

Policy information about availability of data

All manuscripts must include a data availability statement. This statement should provide the following information, where applicable:

- Accession codes, unique identifiers, or web links for publicly available datasets
- A list of figures that have associated raw data
- A description of any restrictions on data availability

The raw mass spectrometry data of peptides eluted from organoid lines MP015 and MP081 have been deposited in the ProteomeXchange Consortium database via the PRIDE [1] partner repository under the dataset identifier PXD018302 (https://www.ebi.ac.uk/pride/). Complete MS search results and lists of peptides eluted from PDAC patients MP015 and MP081 are shown in Supplementary Datasets 1 and 2. The complete datasets generated during and/or analyzed during the current study are not publicly available due to protection of intellectual property by the University of Texas, or for the purposes of protection of patient identification under The Health Insurance Portability and Accountability Act (HIPAA). The corresponding authors will make data from these datasets available upon reasonable request. Publicly available datasets include GTex Portal database (https://www.gtexportal.org/home/) and TCGA database (https://portal.gdc.cancer.gov/). Mascot 2.6.2 was used for protein identification. It searched the following databases: SwissProt Human protein database (2018 September, https://www.uniprot.org/) and the union of human coding sequence (CDS) from Ensembl (release 95) and GENCODE (release 29).

Field-specific reporting

Please select the one below that is the best fit for your research. If you are not sure, read the appropriate sections before making your selection.

[x] Life sciences [ ] Behavioural & social sciences [ ] Ecological, evolutionary & environmental sciences

For a reference copy of the document with all sections, see nature.com/documents/nr-reporting-summary-flat.pdf

Life sciences study design

All studies must disclose on these points even when the disclosure is negative.

Sample size	Since the purpose of the study was to discover any potentially therapeutic PDAC tumor-associated antigen targets, statistical methods were not used to analyze the number of PDAC patients on which to perform the mass spectrometry-based immunopeptidome analysis. Every patient has a unique immunopeptidome, after analyzing over 35 samples we identified 12 unique TAAs of which only one was shared between 2 patients. We decided to move forward with VGLL1 since it had a good safety profile and was shared between two PDAC patients.
Data exclusions	No individual patient data was excluded from the immunopeptidome analysis, in that all peptides were screened as potential therapeutic targets. For all other assays, we did not exclude any samples. If a cell line was HLA-A*0101-negative, it was transduced to express it. If an A*0101-positive cell line did not express VGLL1 protein, then cells were either pulsed with the VGLL1 peptide or it was used as a negative control cell line in Chromium-51 release assays.
Replication	Mass spectrometry analysis was performed at least twice for each VGLL1 peptide target-expressing cell line (MP015-Org, MP081-Org, and Panc10.05): once or twice using discovery phase MS and once using targeted MS with an isotope-labeled synthetic peptide standard. VGLL1 CTL generation against the LSELETPGKY peptide target was successful in 3 of 3 PBMC donors (2 healthy and one PDAC patient). Chromium-51 release assays to assess VGLL1-CTL killing were repeated 2 to 4 times with representative results shown. Western blot analysis was performed 3 times and all attempts at replication were successful.
Randomization	Our study did not involve any comparisons between treatment groups that would require randomization. It used a patient screen to identify VGLL1 as a T-cell target and demonstrated that VGLL1-specific T cells can kill cells expressing the VGLL1 peptide expressed on HLA-A*0101.
Blinding	Blinding was not necessary since our study does not perform comparisons between groups. Since PBMCs were collected and analyzed from one PDAC patient to isolate VGLL1-reactive T cells, there was no reason for blinding.

Reporting for specific materials, systems and methods

We require information from authors about some types of materials, experimental systems and methods used in many studies. Here, indicate whether each material, system or method listed is relevant to your study. If you are not sure if a list item applies to your research, read the appropriate section before selecting a response.

Materials & experimental systems

n/a	Involved in the study	
[ ]	[x]	Antibodies
[ ]	[x]	Eukaryotic cell lines
[x]	[ ]	Palaeontology
[x]	[ ]	Animals and other organisms
[ ]	[x]	Human research participants
[ ]	[x]	Clinical data

Methods

n/a	Involved in the study	
[x]	[ ]	ChIP-seq
[ ]	[x]	Flow cytometry
[x]	[ ]	MRI-based neuroimaging

Antibodies

Antibodies used	The following flow cytometry antibodies were used:<br>US Biological, Cat #: H6098-06A, Anti-human HLA-class A1, A36 (Biotin), Lot: L13082157<br>BD Biosciences, Cat#: 349024, Streptavidin APC, Lot: 8248926<br>BioLegend, Cat#: 344722, APC-conjugated CD8 antibody, clone SK1, lot: B215113<br><br>The following Western blot antibodies were used:<br>Origene Technologies, Cat#:TA322329, Anti-VGLL1 rabbit polyclonal antibody, Lot.: Unknown<br>Abcam, Cat#: ab8227, Rabbit polyclonal anti-beta actin antibody, Lot: GR3224364-1<br><br>HLA class I Immunoprecipitation:<br>Cells were lysed in 1% Triton-X 100 Lysis buffer (PBS PH7.4 to 7.6) in presence of Iodoacetamide (1uM; Cat.I1149-5G), 1uM Leopeptine, 1uM PMSF (Phenylmethylsulfonyl fluoride), Sigma), Halt Protease Inhibitor cocktail and 5 uM EDTA, (Thermo Fisher Scientific Cat. 1861279, lot., SA245363A)). Fifty milligram of total protein pre-cleared with protein A/G ultralink resin (cat. 53133, lot TJ275214) for 4 hours and immunoprecipeted with anti-HLA A,B,C monoclonal antibody (clone: W6/32; Cat 311402, lot B134945) at a ratio 1:1000 (AB:protein) for o/n at 4C. After washing three times in cold PBS (PBS PH7.4 to 7.6), MHC-I bound peptides were eluted by using column with 0.1N acetic acid 1ml each total five times. Before giving to MDACC Mass spectrometry analysis (core facility) for peptide detections, 30ul of each fraction was checked by WB using anti-HLA A antibody (ABGENT, cat-ALS16916).
Validation	The antibodies used are commercially available and were validated by the company for species reactivity and application. We further validated their results by including both a negative and positive control samples to confirm the specificity of the antibody to bind to the target of interest. Additionally, all the experiments were completed in multiples for reproducibility and validation of specific binding.

Eukaryotic cell lines

Policy information about cell lines

Cell line source(s)	ATCC, Panc10.05<br>Cold Spring Harbor Labs, hMIA2D also known as MP015<br>MDADCC, MP081<br>Sigma-Aldrich, BXPC3<br>ATCC, CAPAN-1<br>Sigma-Aldrich, UBCL-1<br>ATCC, HT1197, HLA-A*0101 negative<br>ATCC, HT1376, HLA-A*0101 negative<br>ATCC, BT20<br>ATCC, HCC1187<br>ATCC, BT549<br>MD Anderson Cancer Center Cell Line Registry (MDACC), MKN74, HLA-A*0101 negative<br>MDACC, GT-5, HLA-A*0101 negative<br>MDACC, OVCAR8<br>MDACC, OVCAR433<br>MDACC, OVCAR5<br>MDACC, EBC-1<br>MDACC, Mel888<br>MDACC, WM793<br>LifeLine Technologies, Primary Airway<br>LifeLine Technologies, Primary Melanocytes<br>LifeLine Technologies, Primary Bladder 00400<br>LifeLine Technologies, Primary Bladder-0420<br><br>Unless stated otherwise, all cell lines are HLA-A*0101 positive.
Authentication	HLA typing was performed on some of the cell lines in this study to confirm haplotype provided by the manufacturer and in the literature. Expression of HLA-A*0101 as indicated by high resolution sequencing or public databases was also confirmed by staining all cell lines with an antibody specific for HLA-A*0101 and performing flow cytometry to confirm surface expression. Cell line expression of VGLL1 protein as predicted by RNA analysis was confirmed by Western blot in all cell lines.<br>ATCC, Panc10.05, DNA HLA Typing<br>Cold Spring Harbor Labs, hMIA2D, DNA HLA Typing<br>MDADCC, MP081, DNA HLA Typing<br>Sigma-Aldrich, BXPC3, DNA HLA Typing<br>ATCC, CAPAN-1 , DNA HLA Typing<br>Sigma-Aldrich, UBCL-1 , DNA HLA Typing

	ATCC, HT1197, DNA HLA Typing<br>ATCC, HT1376, DNA HLA Typing<br>ATCC, BT20 , DNA HLA Typing<br>ATCC, HCC1187 , DNA HLA Typing<br>ATCC, BT549 , DNA HLA Typing<br>MDADCC, MKN74 , DNA HLA Typing<br>MDADCC, GT-5 , DNA HLA Typing<br>MDACC, OVCAR8, DNA HLA Typing<br>MDACC, OVCAR433, DNA HLA Typing<br>MDACC, OVCAR5, DNA HLA Typing<br>MDACC, H1975 , DNA HLA typing<br>MDACC, EBC-1, DNA HLA typing<br>MDACC, Mel888 , DNA HLA typing<br>MDACC, WM793, DNA HLA Typing<br>LifeLine Technologies, Primary Airway, DNA HLA typing<br>LifeLine Technologies, Primary Melanocytes, DNA HLA typing<br>LifeLine Technologies, Primary Bladder 00400, DNA HLA typing<br>LifeLine Technologies, Primary Bladder-0420, DNA HLA typing
Mycoplasma contamination	Panc10.05 Mycoplasma tested, Negative<br>MP015 Mycoplasma tested, Negative<br>hMIA2MD Mycoplasma tested, Negative<br>BXPC3 Mycoplasma tested, Negative<br>CAPAN-1 Mycoplasma tested, Negative<br>UBCL-1 Mycoplasma tested, Negative<br>HT1197 Mycoplasma tested, Negative<br>HT1376 Mycoplasma tested, Negative<br>BT20 Mycoplasma tested, Negative<br>HCC1187 Mycoplasma tested, Negative<br>BT549 Mycoplasma tested, Negative<br>MKN74 Mycoplasma tested, Negative<br>GT-5 Mycoplasma tested, Negative<br>OVCAR8 Mycoplasma untested<br>OVCAR433 Mycoplasma untested<br>OVCAR5 Mycoplasma untested<br>H1975 Mycoplasma tested, Negative<br>EBC-1 Mycoplasma tested, Negative<br>Mel888, Mycoplasma tested, Negative<br>WM793 Mycoplasma tested, Negative<br>Primary Airway Mycoplasma tested, Negative<br>Primary Melanocytes Mycoplasma tested, Negative<br>Primary Bladder 00400 Mycoplasma tested, Negative<br>Primary Bladder-0420 Mycoplasma tested, Negative
Commonly misidentified lines<br>(See ICLAC register)	None tested.

Human research participants

Policy information about studies involving human research participants

Population characteristics	Non-interventional research studies to generate VGLL1-specific T cells were performed using PBMC samples from:<br>1 Pt with PDAC (HLA-A*0101 positive, M, 55yo, pancreatic adenocarcinoma)<br>2 healthy donors (HLA-A*0101-positive, healthy donor information was blinded due to HIPPA)
Recruitment	Pancreatic adenocarcinoma patients and healthy donors were recruited following standard consent per protocol. We studied healthy donors that were HLA-A*0101 positive, since only A*0101-positive patients would possess VGLL1 peptide-reactive T cells (the VGLL1 peptide is restricted to A*0101).
Ethics oversight	Oversight was provided by the MD Anderson Cancer Center Clinical Trials Institutional Review Board

Note that full information on the approval of the study protocol must also be provided in the manuscript.

Clinical data

Policy information about clinical studies

All manuscripts should comply with the ICMJE guidelines for publication of clinical research and a completed CONSORT checklist must be included with all submissions.

Clinical trial registration	N/A – Both Minimal Risk Lab (Specimen Collection) protocols
Study protocol	PA14-0138, PA14-0105 (MDACC protocol numbers)
Data collection	Patient and Healthy donors underwent standard leukapheresis procedures at MD Anderson Cancer Center facility.
Outcomes	N/A: Minimal risk lab protocol (to collect cancer pt PBMC), Minimal risk lab protocol (to collect healthy donor PBMC)

Flow Cytometry

Plots

Confirm that:

- [x] The axis labels state the marker and fluorochrome used (e.g. CD4-FITC).
- [x] The axis scales are clearly visible. Include numbers along axes only for bottom left plot of group (a 'group' is an analysis of identical markers).
- [x] All plots are contour plots with outliers or pseudocolor plots.
- [x] A numerical value for number of cells or percentage (with statistics) is provided.

Methodology

Sample preparation	For HLA-A*01:01 staining, each sample was first washed in FACS Buffer. After washing, the samples were then incubated on ice in 50uL of buffer with 10uL of anti-HLA-A*0101 antibody for 30 minutes. The antibody was then washed off, and the cells were re-suspended in 50uL of FACS buffer. A total of 10uL of streptavidin was added and the cells were incubated on ice for another 30 minutes. After the incubation period, the cells were washed and re-suspended in 180uL of FACs buffer for running.<br>For CTL staining, cultured cells were stained with VGLL1(231-240) peptide/HLA-A*0101–PE-conjugated custom tetramer (Fred Hutchinson Cancer Research Center), washed and then stained with APC-conjugated CD8 antibody.
Instrument	For tumor cell staining, the BD FACSCanto™ II flow cytometer was used. It comes with with 3 lasers and is able to detect up to 8 colors, and includes FACSDIVA software (version 8.0.1). T-cells were analyzed by flow cytometry (LSRFortessa X-20 Analyzer). CD8 and<br>tetramer double-positive cells were sorted by ARIA II.
Software	FACSDIVA Version 8.0.1 and FlowJoTM Version 10.5.3 software were used for flow cytometric analysis.
Cell population abundance	VGLL1-specific T cells were sorted by double staining with CD8 and VGLL1(231-240) peptide/HLA-A*0101–PE-conjugated custom tetramer and populations of tetramer-positive cells were analyzed in FloJo (version 7.6.5).
Gating strategy	Gates were drawn to sort VGLL1 tetramer-positive CD8+ T cells as indicated in Figures 4 and S18.

- [x] Tick this box to confirm that a figure exemplifying the gating strategy is provided in the Supplementary Information.
